# A GluN2B disease-associated variant promotes the degradation of NMDA receptors *via* autophagy

**DOI:** 10.1016/j.jbc.2026.111147

**Published:** 2026-01-10

**Authors:** Taylor M. Benske, Marnie P. Williams, Pei-Pei Zhang, Adrian J. Palumbo, Ting-Wei Mu

**Affiliations:** Department of Physiology and Biophysics, Case Western Reserve University School of Medicine, Cleveland, Ohio, USA

**Keywords:** ER-phagy, LC3-interacting region (LIR), NMDAR, proteostasis, CCPG1

## Abstract

N-methyl-D-aspartate receptors (NMDARs) are essential for excitatory neurotransmission, and missense mutations can severely disrupt their function. Pathogenic variants often lead to proteostasis defects, including improper folding, impaired assembly, and reduced trafficking to the plasma membrane, ultimately compromising the physiological function of NMDARs and thereby contributing to neurological diseases. However, mechanisms by which the proteostasis network recognizes and degrades aggregated, misfolded, and trafficking-deficient pathogenic NMDARs remain poorly understood. Here, we demonstrate that the R519Q GluN2B variant is retained in the endoplasmic reticulum (ER) and fails to traffic to the cell surface to form functional NMDARs. Pharmacological and genetic inhibition of autophagy resulted in the accumulation of this variant, indicating that it is degraded by the autophagy-lysosomal proteolysis pathway. Since the GluN2B subunit has a cytosolic LC3-interacting region (LIR) motif, disruption of the LIR motif *via* mutagenesis similarly impairs the autophagic clearance of this variant. Furthermore, we demonstrate that this variant is recognized by the ER-phagy receptor CCPG1 and that the LIR domain plays a facilitative role in strengthening this interaction. Our results provide a novel molecular mechanism for the ER-to-lysosome-associated degradation of NMDAR variants and identify a pathway for targeted therapeutic intervention for neurological disorders with dysfunctional NMDARs.

*N*-methyl-D-aspartate receptors (NMDARs) are ionotropic glutamate receptors that mediate excitatory neurotransmission, support synaptic development and plasticity, and maintain the excitation-inhibition balance in the central nervous system (1). These heterotetrameric receptors assemble from two obligatory, glycine-binding, GluN1 subunits and two GluN2(A-D) or GluN3(A-B) subunits, encoded by seven *GRIN* genes (2). In the human forebrain, NMDARs predominantly contain GluN2A and/or GluN2B subunits, likely forming triheteromeric receptors (GluN1/GluN2A/GluN2B) (3, 4, 5, 6). Structurally, GluN subunits consist of four distinct domains: an extracellular amino-terminal domain (ATD), the ligand-binding domain (LBD), which is formed by two segments S1 and S2, the transmembrane domain (TMD), consisting of three helices and one reentrant loop, and an intracellular carboxyl-terminal domain (CTD) (Fig. 1*A*) (7, 8). The glutamate-binding GluN2 subunits impart unique biophysical and pharmacological properties to the NMDARs which underlie subunit-specific regional and temporal expression patterns (9, 10), with GluN2B being abundantly expressed prenatally/embryonically, and GluN2A expression predominating at postnatal, mature synapses (11). Proper assembly and intracellular processing of these subunits are essential for functional expression at the synapse.

**Figure 1:**
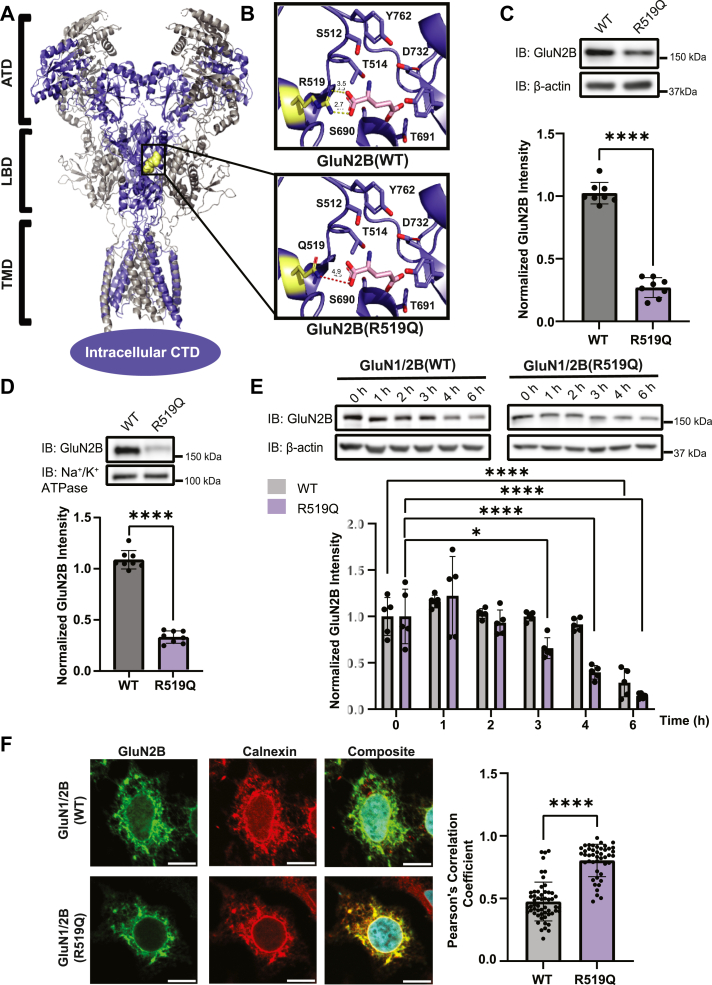
**Molecular characterization of the disease-associated variant GluN2B_R519Q**. *A*, structure of the rat tetrameric GluN1_GluN2B NMDA receptor in complex with glutamate, with the GluN1 subunit in *gray*, and the GluN2B subunit in blue (PDB:9ARI) (42). The amino-terminal domain (ATD), ligand-binding domain (LBD), and transmembrane domain (TMD) are shown in ribbon format with the R519 residue selected for study in *yellow* spheres. *B*, structural model displaying residues involved in the glutamate binding site of the WT GluN2B subunit are represented as sticks, with the glutamate ligand in *pink* (*upper*). *In silico* mutagenesis of the R519 residue to a glutamine eliminates the electrostatic interaction with glutamate (*lower*). *C*, effects of R519Q on total GluN2B protein expression levels 48 h post transient transfection of HEK293T with GluN1 and GluN2B constructs at a 1:1 ratio to express WT or GluN2B_R519Q variant NMDARs. β-actin serves as the soluble total protein loading control (n = 7). *D*, surface biotinylation assay to monitor the influence of the R519Q DAV on the surface expression of NMDARs 48 h post transient transfection. Na^+^/K^+^ ATPase served as a membrane protein loading control (n = 8). *E*, HEK293T cells stably expressing WT or R519Q GluN2B NMDARs were subjected to cycloheximide (100 μg/ml) for the indicated times to determine the stability and rate of degradation. β-actin serves as the soluble total protein loading control (n = 5). Statistical significance was determined by a two-way repeated measures analysis of variance (ANOVA) followed by a *post hoc* Tukey test for comparison in multiple groups. *F*, immunofluorescence images of GluN2B (*green*) and calnexin (*red*), an ER marker to assess ER accumulation (scale bar = 10 μm). Pearson’s coefficients are reported (n > 30 cells) and statistical significance was determined using a Mann-Whitney test. All data are normalized to the appropriate loading control, and data are presented as mean ± SD. Statistical significance was determined using an unpaired two-tailed Student’s *t* test between two groups for (*C*) and (*D*). Significance level defined as ∗*p* < 0.05, ∗∗∗∗*p* < 0.0001.

Following their synthesis, NMDAR subunits fold and assemble in the endoplasmic reticulum (ER) into functional receptors and must pass stringent quality control checkpoints before anterograde transport to the Golgi apparatus and plasma membrane (12, 13, 14, 15, 16). Within the ER, chaperone proteins coordinate folding, assembly, glycosylation, and disulfide bond formation to promote native protein conformation. Ligand binding serves as a critical quality control step for forward trafficking of NMDARs, underscoring the importance of the structural integrity within the LBD (17, 18, 19, 20). Missense mutations can exacerbate the inefficiency of folding and assembly (21), leading to the accumulation of misfolded proteins in the ER. This accumulation can drive ER volume expansion, reduce folding capacity, induce ER stress, and activate the unfolded protein response (UPR) (22, 23, 24).

Whole-genome sequencing has identified numerous disease-associated variants (DAVs) in *GRIN* genes in patients with neurodevelopmental disorders, including autism spectrum disorder, intellectual disability, developmental delay, and epilepsies (25, 26, 27, 28). Patients often present additional symptoms such as hypotonia, movement disorders, behavioral abnormalities, and gastrointestinal issues (ClinVar). *GRIN* DAVs are typically *de novo* and heterozygous, with loss-of-function mutations exhibiting haploinsufficiency (25, 27). DAVs in the GluN2B subunit cluster in the LBD and ion pore, highlighting the critical role of these domains in receptor activity (29). *GRIN* genes are highly intolerant to variation, with *GRIN2B* ranking among the top 1.07% of most constrained genes in the human genome (30, 31). Despite this, *GRIN* genes remain highly polymorphic, and many DAVs remain uncharacterized. Altered surface trafficking and synaptic localization due to NMDAR DAVs are thought to contribute to the pathophysiology, though the mechanisms remain poorly understood. Among these variants, the GluN2B R519Q disease-associated variant (ClinVar Variation ID: 981287), located in the LBD and reported in multiple individuals with neurodevelopmental delay and intellectual disability, was selected to investigate how GluN2B DAVs affect NMDAR folding, trafficking, and engagement of cellular proteostasis pathways.

Maintenance of cellular proteostasis relies on the efficient clearing of terminally misfolded, aggregated, and short-lived proteins through ER-associated degradation (ERAD), in which misfolded substrates are reterotranslocated to the cytosol and degraded by the ubiquitin-proteasome system. However, large misfolded or aggregated proteins can be resistant to ERAD and are instead sequestered in ER subdomains and cleared through ER-to-lysosome-associated degradation (ERLAD). ER-phagy (reticulophagy) provides a selective autophagy pathway for removing misfolded, aggregated, or ERAD-resistant proteins. ER-phagy relies on ER-resident, membrane-bound receptors containing LC3-interacting regions (LIR), which recruit ATG8 family proteins (LC3/GABARAP) and couple the ER to the autophagy machinery (32, 33). These ER-phagy receptors include FAM134 paralogs (A,B,C), RTN3L, ATL3, SEC62, CCPG1, and TEX264 (34, 35), each occupying distinct ER subdomains: FAM134b localizes at the curved edges of ER sheets (36), RTN3, ATL3 and TEX264 are in the ER tubules, and CCPG1 and SEC62 localize within the ER sheets, particularly near insoluble protein aggregates under stress conditions (37, 38). By mediating cargo segregation, membrane shaping, and delivery of folding-deficient proteins and damaged ER regions for degradation, ER-phagy receptors ensure ER quality control through ERLAD (39).

Although the general effects of DAVs within the GluN2B polypeptide sequence have been extensively characterized at the level of NMDAR function, much less is known about how the resulting aberrant proteins interface with ER proteostasis networks and degradation pathways. Here, we used HEK293T cells expressing NMDARs containing the R519Q variant to evaluate how this variant influences the biogenesis of GluN2B and clearance through proteolytic pathways. Characterization of this variant revealed prominent folding and trafficking defects, as evidenced by its retention within the ER. Furthermore, genetic and pharmacological inhibition of autophagy demonstrated that the GluN2B subunit is degraded *via* the autophagy-lysosomal pathway and engages the ER-phagy receptor CCPG1, whose interaction is strengthened by the C-terminal LIR motif within the GluN2B subunit. Together, these findings support a model in which certain aggregation-prone NMDAR variants preferentially direct receptor subunits to the autophagy-lysosome pathway as a key component of their pathogenic mechanism.

## Results

### The R519Q GluN2B variant reduces the protein expression and stability of NMDARs and results in ER retention

To assess the clinical and structural relevance of DAVs within the GluN2B LBD, we identified 160 single-nucleotide polymorphisms resulting in missense mutations present in the S1/S2 segments from NIH ClinVar (https://www.ncbi.nlm.nih.gov/clinvar/, last accessed December 1, 2025). Of these, 53% are classified as pathogenic or likely pathogenic, while 44% have conflicting reports or uncertain significance (Fig. S1*A*, Table S1). We next used Rhapsody, an *in silico* saturation mutagenesis tool, to predict the pathogenicity of these DAVs (40), which identified 64% of GluN2B LBD variants are deleterious based on evolutionary conservation, structural context, and physicochemical impacts (Table S1, Fig. S1*B*). Among these, the R519Q variant was selected for detailed study due to the high conservation of the R519 residue and its clinical significance, with four unique ClinVar submissions linking it to intellectual disability, neurodevelopmental disorder, and developmental epileptic encephalopathy, 27 (DEE27) (41) It also has a severe deleterious Rhapsody score (0.704) and has not been extensively characterized, despite three alternative amino acid substitutions (R519G, R519L, and R519Q) and truncation due to a nonsense mutation at this site.

This study focuses on the R519 residue of the GluN2B subunit, whose position is highlighted in a structural model of an NMDAR comprised of GluN1 and GluN2B subunits (Fig. 1*A*). The arginine residue at position 519 of the GluN2B subunit directly interacts with the glutamate ligand, forming a salt bridge that is critical for agonist potency and stability of the protein-ligand complex (42). Substitution of this arginine with a glutamine, as modeled by *in silico* mutagenesis (Fig. 1*B*), removes the positive charge in the binding pocket, likely disrupting the salt bridge, destabilizing the complex and triggering protein misfolding, reduced surface trafficking, and excessive degradation. NMDARs require assembly of GluN1 and GluN2 subunits to form functional receptors. Therefore, to evaluate the effects of the R519Q variant on the protein levels of NMDARs, we co-expressed wild type (WT) or R519Q GluN2B subunits with GluN1 subunits in HEK293T cells, using a 1:1 ratio of cDNA, in order to express exogenous receptors. Cells in culture were supplemented with MK-801, a potent NMDAR antagonist, to prevent glutamate-induced excitotoxicity, and harvested 48 h after transfection. Western blot analysis of total proteins revealed that the R519Q expression was reduced drastically, with a 3.8-fold decrease compared to WT GluN2B (Fig. 1*C*). Given that the surface expression of NMDARs is critical for their physiological function, we carried out a surface biotinylation assay and demonstrated that the surface expression of the R519Q variant was similarly reduced to 30% of the WT levels (Fig. 1*D*). Additional pathogenic mutations within the GluN2B LBD E413G, C461F, and T514A, were investigated and found to modulate surface expression of NMDARs (Fig. S1*C*), establishing this phenotype is a common feature of pathogenicity. Interestingly, the T514A variant increased the surface expression of the GluN2B subunit (Fig. S1*C*), suggesting that it does not promote misfolding and may stabilize the receptor for trafficking. T514A has a modest pathogenicity score of 0.594 (Table S1).

Next, we performed a cycloheximide (CHX) chase assay to determine the half-lives of the WT and R519Q GluN2B subunits and assess their stability. HEK293T cells stably expressing NMDARs containing either WT or R519Q GluN2B subunits were treated with CHX, a potent inhibitor of protein synthesis, and chased for the indicated time. The R519Q variant demonstrates a faster decay time, with significant decrease in abundance starting after 3 h, relative to the WT which did not show significant decreases until after 6 h (Fig. 1*E*). This reduced stability of the R519Q variant correlates with their observed steady-state protein levels and indicates faster degradation kinetics.

NMDAR subunits must properly fold and assemble within the ER before they can traffic to the plasma membrane. Therefore, since the R519Q variant demonstrated a pronounced decrease in surface expression, we asked whether this variant was retained in the ER. We used immunocytochemistry confocal imaging to determine the colocalization of the WT and R519Q variant with the endogenous ER-resident protein calnexin. The R519Q variant displayed substantial overlap and exhibited greater Pearson’s correlation coefficients compared to the WT GluN2B subunit (Fig. 1*F*). This indicates that the R519Q variant is more frequently retained within the ER, likely failing quality control measures and thus preventing its incorporation into functional receptors able to traffic to the plasma membrane. Collectively, our results indicate that the R519Q variant perturbs NMDAR proteostasis by reducing the stability of the GluN2B subunit, resulting in ER retention and reduced total and surface expression, which may underlie the pathophysiology of *GRIN2B*-related neurodevelopmental disorders.

### Pharmacological and genetic inhibition of proteolysis pathways demonstrate that the R519Q DAV drives GluN2B degradation toward autophagy

**Figure 2:**
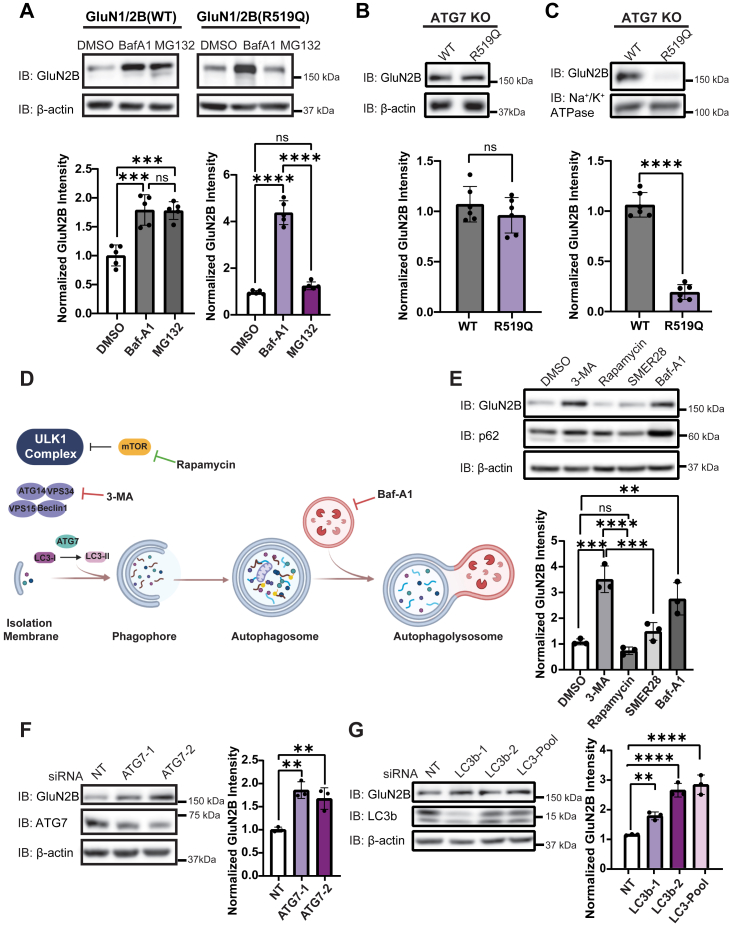
**The GluN2B subunit undergoes degradation *via* autophagy**. *A*, inhibition of the proteasome with MG132 (10 μM) and the lysosome with Bafilomycin-A1 (1 μM) for 6 h effect on the GluN2B subunit in HEK293T cells stably expressing recombinant WT or R519Q NMDARs. β-actin serves as the soluble total protein loading control (n = 5). *B*, effects of R519Q on total GluN2B protein expression levels 48 h post transient transfection with GluN1 and GluN2B constructs at a 1:1 ratio in ATG7 KO HEK293T to express WT or GluN2B_R519Q variant NMDARs. β-actin served as the soluble total protein loading control. (n = 6). *C*, surface biotinylation assay to monitor the influence of the R519Q DAV on the surface expression of NMDARs expressed in ATG7 KO HEK293T 48 h post transient transfection. Na^+^/K^+^ ATPase served as a membrane protein loading control (n = 6). *D*, schematic of macroautophagy pathway, including induction, nucleation and formation of the isolation membrane, elongation, autophagosome formation, and fusion with the lysosome forming the autophagolysosome. Rapamycin is shown to inhibit mTOR, which results in autophagy activation, while 3-MA and Baf-A1 inhibit autophagy at different stages as shown. *E*, HEK293T cells stably expressing R519Q NMDARs were treated with autophagy activators (SMER28 10 μM, Rapamycin 100 nM) and inhibitors of autophagy (3-MA 50 mM and Baf-A1 20 nM) for 24 h. Changes to total GluN2B expression were monitored *via* immunoblot and p62 was used as a marker for autophagic flux. β-actin serves as the soluble total protein loading control (n = 3). *F*, siRNA knockdown of ATG7 effect on R519Q variant GluN2B expression and immunoblot was performed 48 h after knockdown. β-actin served as the soluble total protein loading control (n = 3). Non-targeting (NT) siRNA was used as a control for each condition, and two independent siRNA constructs were used to knockdown gene expression. *G*, siRNA knockdown of LC3b effects on R519Q variant GluN2B expression. Immunoblot performed 48 h after knockdown. β-actin served as the soluble total protein loading control (n = 3). Non-targeting siRNA was used as a control for each condition, and two independent siRNA constructs were used to knockdown gene expression. LC3-Pool contains two unique siRNAs for each of the LC3a, LC3b, and LC3c isoforms. All data are normalized to the appropriate loading control, and data are presented as mean ± SD. Statistical significance was determined using an unpaired two-tailed Student’s *t* test between two groups or an analysis of variance (ANOVA) followed by a *post hoc* Dunnett’s test for comparison in multiple groups. Significance level defined as ns, not significant, ∗∗*p* < 0.01, ∗∗∗*p* < 0.001, ∗∗∗∗*p* < 0.0001.

Given that the R519Q variant exhibited reduced stability and increased ER retention, we sought to elucidate how proteolytic pathways mediate its clearance. We pharmacologically inhibited lysosomal degradation with bafilomycin A1 (Baf-A1), a potent V-type ATPase inhibitor, and proteasomal degradation with MG132, a potent proteasome inhibitor. HEK293T cells stably expressing NMDARs with WT or R519Q GluN2B were treated for 6 h with 1 μM Baf-A1 or 10 μM MG132. In WT-expressing cells, treatment with both Baf-A1 and MG132 resulted in a modest 1.79-fold accumulation of GluN2B compared to the DMSO control, suggesting that both pathways are utilized. In contrast, cells expressing the R519Q NMDARs displayed a striking 4.4-fold increase upon treatment with Baf-A1, while treatment with MG132 did not significantly affect GluN2B expression (Fig. 2*A*). These findings represent a 2.5-fold higher accumulation of the R519Q variant subunit relative to the WT after Baf-A1 treatment, suggesting that the R519Q variant predisposes the GluN2B subunit to clearance by lysosomal degradation (Fig. S1*D*). In contrast, although the E413G, C461F, and T514A DAVs accumulated significantly following treatment with either Baf-A1 or MG132 (Fig. S1*E–G*), preferential lysosomal degradation was less evident for these variants than for R519Q. Among them, the E413G DAV exhibits a partial preference for the lysosomal degradation.

Building on these findings, we investigated whether autophagy contributes to the preferential degradation of the R519Q DAV by employing HEK293T cells with a CRISPR-mediated knockout of ATG7, an essential autophagy protein responsible for initiating autophagosome formation through LC3b lipidation. This approach allows us to examine the sustained effects of chronic autophagy depletion on GluN2B proteostasis while accounting for any degradation driven by transient transfection-induced cell stress. We first validated the ATG7 KO line using an immunoblot assay to confirm the near-complete depletion of ATG7 protein (Fig. S2*A*). As expected, ATG7 expression was severely diminished, and levels of p62/SQSTM1, a canonical autophagy substrate, were significantly elevated. These findings indicate impaired autophagic flux and confirm that this model is suitable for our investigations. To assess the impact of autophagy deficiency on GluN2B stability, we exogenously expressed NMDARs containing WT or R519Q GluN2B subunits in the ATG7 KO cells. Remarkably, immunoblot analysis revealed that the R519Q variant was expressed at levels comparable to the WT in the absence of autophagy (Fig. 2*B*). We next sought to determine whether the surface expression of the R519Q variant could similarly be rescued in ATG7 KO cells. Intriguingly, surface biotinylation assays demonstrated that the R519Q variant was only able to traffic to the plasma membrane around 30% relative to the WT (Fig. 2*C*), despite the comparable total protein levels relative to WT (Fig. 2*B*). These findings indicate that while the inhibition of autophagy can restore the intracellular R519Q GluN2B pool, it does not influence their ability to overcome folding defects and assemble into functional receptors for their anterograde trafficking.

As the ATG7 KO cells lack macroautophagy but not all lysosomal or proteasomal degradation, we next investigated whether there was compensation in the degradation of the GluN2B subunit. Thereby, we treated ATG7 KO cells expressing either WT or R519Q NMDARs with 1 μM Baf-A1 or 10 μM MG132 for 6 h. Immunoblot analysis showed no significant accumulation of either the WT or the R519Q variant upon inhibition of either proteolytic pathway (Fig. S2*B* and *C*). Furthermore, immunofluorescence confocal microscopy revealed an increase in the GluN2B/calnexin (M1) colocalization of the R519Q variant in comparison to the WT, indicating autophagic inhibition does not relieve folding defects and ER retention (Fig. S2*D*).

To further confirm that the observed accumulation of the R519Q variant following Baf-A1 treatment was a direct result of autophagic inhibition, we used additional pharmacological modulators of autophagy that target different stages to monitor the effects on GluN2B expression (Fig. 2*D*). HEK293T cells stably expressing NMDARs containing R519Q variants were treated for 24 h with autophagy inhibitors and activators. Inhibition of autophagy with 3-methyladednine (3-MA; 50 mM), a PI3K inhibitor for VPS34, resulted in a 3.3-fold increase in total GluN2B levels, similar to the Baf-A1 treatment (20 nM). Conversely, autophagy was activated with rapamycin (100 nM), an mTOR inhibitor, or by SMER28 (10 μM), a small molecule that induces autophagy in an mTOR-independent fashion, and neither significantly altered the GluN2B expression. p62 expression was assessed as an indicator of autophagic flux, as it is selectively degraded within autolysosomes and therefore accumulates when autophagy is impaired and decreases during autophagy activation (Fig. 2*E*). Interestingly, surface biotinylation with autophagy modulators revealed that 24 h treatment with 3-MA increased the surface expression of the R519Q variant, while Baf-A1, rapamycin, and SMER28 did not influence surface trafficking. Of note, the effect of 3-MA treatment may be independent of its canonical inhibition of class III PI3Ks, as there was no observable change to LC3b lipidation (Fig. S3*A*). Finally, in an effort to utilize genetic depletion of core components of macroautophagy machinery gene products to demonstrate a role in the degradation of NMDARs containing a pathogenic variant, we performed a siRNA-mediated knockdown ATG7 and LC3b using two unique siRNAs. Consistent with our ATG7 KO cell line, knockdown of ATG7 expression resulted in the accumulation of R519Q variant GluN2B subunit by 76% compared to the non-targeting control (Fig. 2*F*, Fig. S3*B*). Additionally, we performed a knockdown of LC3b, the most predominant LC3 isoform and extensively described for canonical macroautophagy readout, which resulted in a 1.5- to 2.5-fold increase in GluN2B expression. Furthermore, we investigated whether the LC3 isoforms LC3a, LC3b, and LC3c, contributed to NMDAR degradation. We utilized a pool of siRNAs containing two unique siRNAs per isoform, leading to a 3.36-fold increase in GluN2B protein levels (Fig. 2*G*, Fig. S3*C*). Taken together, these results implicate canonical macroautophagy in the degradation of the R519Q DAV through ATG7-and LC3-dependent clearance *via* the lysosome.

### The R519Q GluN2B variant is targeted toward the lysosome *via* interactions with the ER-phagy receptor CCPG1

**Figure 3:**
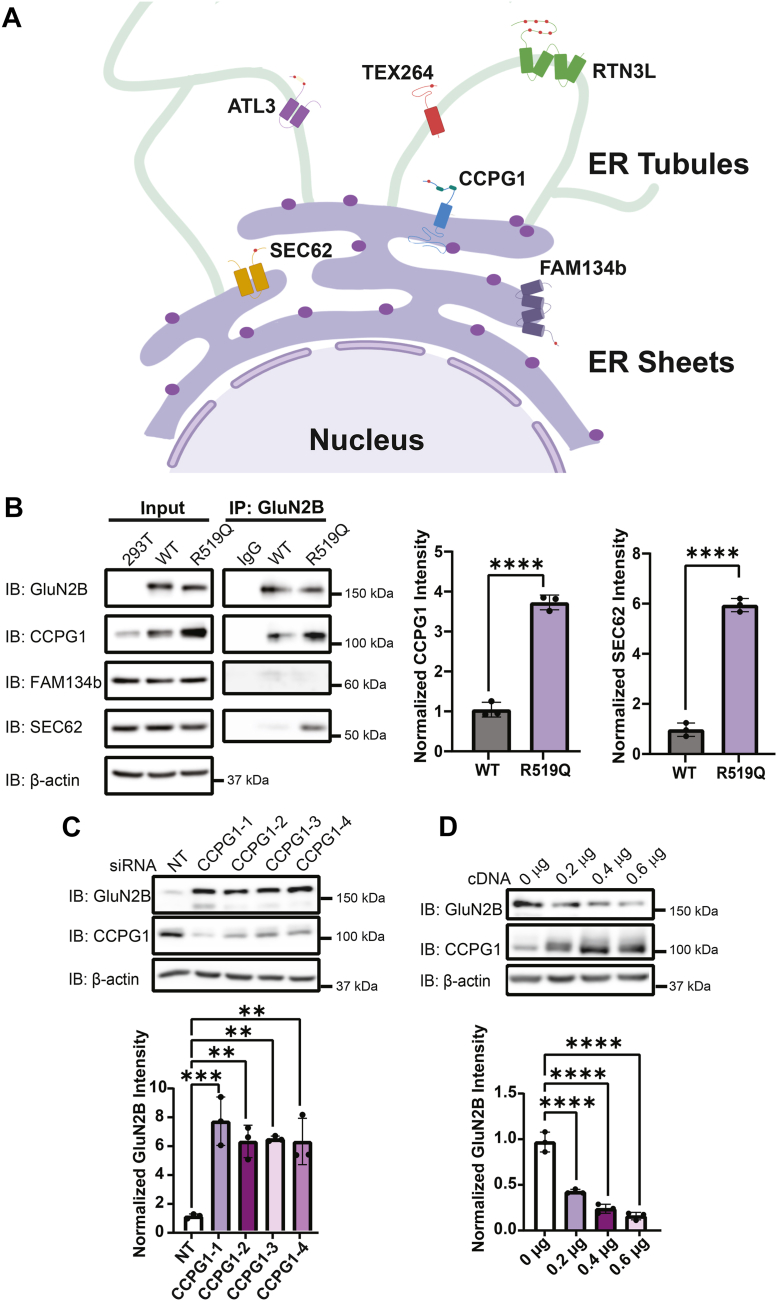
**The ER-phagy receptor CCPG1 interacts with the R519Q GluN2B variant and promotes its degradation through ER-to-lysosome-associated degradation**. *A*, essential membrane-bound ER-phagy receptors. CCPG1, SEC62, and FAM134b are generally located in the ER sheets, while RTN3L, TEX264, and ATL3 are in the ER tubules. All ER-phagy receptors contain at least one LIR, represented as red circles, but induce degradation of distinct ER subdomains and client proteins. *B*, co-immunoprecipitation of HEK293T cells exogenously expressing R519Q GluN2B variants was done to measure interaction with ER-phagy receptors present in ER sheets CCPG1, FAM134b, and SEC62. Cells were treated with Baf-A1 (20 nM for 24 h), 24 h post transient transfection to enrich autophagy proteins for interaction. Quantification of the ratio of the protein of interest to GluN2B post co-IP is shown in the right panel (n = 3). *C*, siRNA knockdown of CCPG1 effects on GluN2B expression in HEK293T cells stably expressing R519Q NMDARs. Immunoblots performed 48 h after knockdown. Non-targeting siRNA was used as a control for each condition, and four independent siRNA constructs were used to knockdown gene expression. β-actin served as the soluble total protein loading control (n = 3). *D*, Dose–response overexpression of CCPG1 cDNA in HEK293T cells stably expressing R519Q GluN2B variants NMDARs. Cells were harvested 24 h after transient transfection. β-actin served as the soluble total protein loading control (n = 3). All data are normalized to the appropriate loading control, and data are presented as mean ± SD. Statistical significance was determined using an unpaired two-tailed Student’s *t* test between two groups or an analysis of variance (ANOVA) followed by a *post hoc* Dunnett’s test for comparison in multiple groups. Significance level defined as ∗∗*p* < 0.01, ∗∗∗*p* < 0.001, ∗∗∗∗*p* < 0.0001.

Folding-defective proteins that cannot traverse the ER membrane for ERAD accumulate within the ER and are delivered to the lysosome *via* interactions with ER-phagy receptors. Because the R519Q variant exhibits ER retention and preferential degradation *via* autophagy, we investigated whether ER-phagy receptors contribute to NMDAR homeostasis. All of the six known mammalian, membrane-bound ER-phagy receptors contain at least one LIR domain that recruits autophagy machinery and facilitates the degradation of ER regions *via* interaction with ATG8 family proteins. CCPG1, SEC62, and FAM134b respond to resolve ER stress and localize to the ER sheets, the site of secretory protein biogenesis, with FAM134b specifically enriched at regions of membrane curvature, whereas tubular ER homeostasis is maintained by RTN3L, TEX264, and ATL3 (Fig. 3*A*).

We performed co-immunoprecipitation assays to assess interactions between the R519Q variant and ER-phagy receptors. Cells were treated 20 nM Baf-A1 for 24 h to enhance autophagy protein interactions. Among ER sheets localized ER-phagy receptors, R519Q displayed robust interactions with CCPG1, while interactions with SEC62 were marginal and those with FAM134b were undetectable, suggesting that CCPG1 represents potentially biologically relevant interactions in this system (Fig. 3*B*). Further analysis of tubular ER-phagy receptors revealed a significant interaction with RTN3L (Fig. S4*C*).

Next, we used siRNA knockdown of the ER-phagy receptors that interacted with the R519Q variant. Remarkably, CCPG1 knockdown induced a ∼6-fold increase in R519Q total protein levels (Fig. 3*C*, Fig. S4*A*), while knockdown of RTN3L also increased the R519Q by a modest ∼2-fold (Fig. S4*D*). Conversely, overexpression of CCPG1 caused a dose-dependent decrease in the R519Q expression, confirming that CCPG1 mediates autophagic clearance of R519Q variants (Fig. 3*D*, Fig. S4*B*). Together, these results indicate that CCPG1 and, to a lesser extent RTN3L, mediate the clearance of accumulated R519Q variants from the ER.

### The highly conserved GluN2B LC3-interacting region (LIR) influences the proteostasis of NMDA receptors

**Figure 4:**
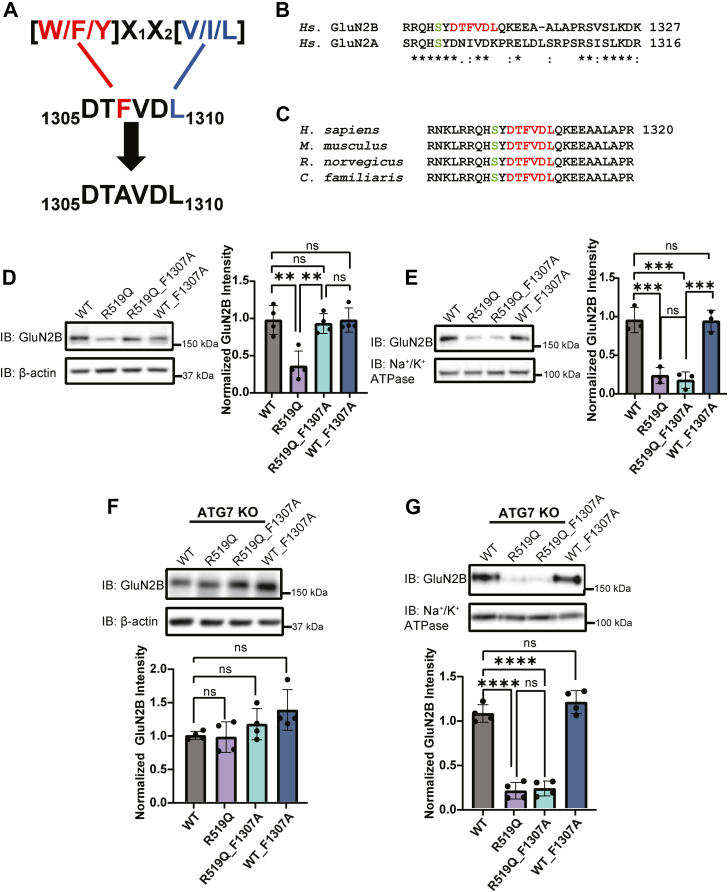
**The GluN2B LC3-interacting region (LIR) motif is highly conserved**. *A*, canonical LC3-interacting motif consensus sequence pointing to the LIR motif identified in the GluN2B subunit and subsequent alanine substitution. Aromatic residue is shown in red, hydrophobic residue in blue. *B*, sequence alignment of *Homo sapiens* GluN2A and GluN2B CTD amino acid sequence in the region of interest. The LIR motif is in red in the GluN2B subunit. The serine residue that is phosphorylated by CAMKII is shown in green for each subunit. Asterisks mark conserved residues, colons mark conserved residues with similar properties, and a period denotes residues that are semi-conservative. *C*, amino acid sequence alignment of mammalian species demonstrating species conservation of the GluN2B CTD region of interest surrounding the LIR motif. *D*, effects of LIR motif disruption on WT, WT_F1307A and R519Q, R519Q_F1307A total GluN2B protein expression levels 48 h post transient transfection of HEK293T cells transfected with a 1:1 ratio of GluN1 and GluN2B constructs to express WT or GluN2B_R519Q variant NMDARs. β-actin served as the soluble total protein loading control (n = 4). *E*, surface biotinylation assay to monitor the influence of the LIR domain disruption on the WT and R519Q DAV surface expression of NMDARs 48 h post transient transfection Na^+^/K^+^ ATPase served as a membrane protein loading control (n = 3). *F*, effects of LIR motif disruption on total expression of GluN2B subunits expressed in ATG7 KO cells. 48 h post transient transfection of GluN1 and GluN2B constructs with a 1:1 ratio of GluN1 and GluN2B constructs to express WT, WT_F1307A, R519Q, R519Q_F1307A variant NMDARs. β-actin served as the soluble total protein loading control (n = 4). *G*, surface biotinylation assay to monitor the influence of the LIR motif disruption on WT and the R519Q variant GluN2B subunits on the surface expression of NMDARs in ATG7 KO cells 48 h post transient transfection. Na^+^/K^+^ ATPase served as a membrane protein loading control (n = 4). All data are normalized to the appropriate loading control, and data are presented as mean ± SD. Statistical significance was determined using an unpaired two-tailed Student’s *t* test between two groups or an analysis of variance (ANOVA) followed by a *post hoc* Tukey test for comparison in multiple groups. Significance level defined as ns, not significant, ∗∗*p* < 0.01, ∗∗∗*p* < 0.001, ∗∗∗∗*p* < 0.0001.

LIR motifs are present in proteins that undergo selective autophagy and have been observed in autophagy cargo receptors, basal autophagy proteins, proteins associated with vesicles, and signaling proteins and receptors that are degraded by selective autophagy. LIR motifs are characterized by the consensus sequence [W/F/Y]X_1_X_2_[L/I/V] (Fig. 4*A*), where the first residue is a bulky aromatic residue, X_1_ and X_2_ can be any amino acid and the fourth residue is a hydrophobic residue (43, 44). We utilized the iLIR database, an online web resource for identifying LIR motif-containing proteins in eukaryotes (45), and discovered that the human GluN2B subunit was predicted to contain an F-type LIR motif within the CTD consisting of the residues DTFVDL at positions 1305 to 1310 (Fig. 4, *A* and *B*). Notably, in F-type LIR domains, the N or C terminus must be flanked by an acidic residue, the residue present at X_1_ must be a valine, cysteine, isoleucine, or glutamic acid. These constraints are in place as F-type LIR motifs demonstrate weaker affinity for ATG8 family proteins (LC3/GABARAP proteins), and the greater number of electrostatic interactions has been shown to compensate for lower affinities (43, 46). Of all the GluN subunits, the GluN2B subunit alone has a predicted canonical LIR motif. Although the elongated CTDs of GluN2A and GluN2B enable similar interactions, such as CAMKII phosphorylation and PSD95 binding (47, 48, 49), sequence alignment of the human GluN2A and GluN2B CTD region containing the LIR motif revealed only 50% conservation. The analogous GluN2A sequence (DNIVDK, residues 1293–1298) lacks LIR motif characteristics (Fig. 4*B*). Furthermore, the GluN2B LIR motif is absolutely conserved across mammalian species, including humans, mice, rats, dogs, and chimpanzees, emphasizing its functional importance to the GluN2B subunit (Fig. 4*C*).

To investigate the role of the LIR motif, we performed alanine substitution at the critical phenylalanine (F1307) residue (Fig. 4*A*) to disrupt the hydrophobic interaction required for binding to LC3b proteins. We co-expressed GluN1 with either WT, WT_F1037A, R519Q, or R519Q_F1307A GluN2B subunits in HEK293T cells. Western blot analysis demonstrated that the R519Q_F1307A disruption of the LIR domain increased the total expression of the GluN2B subunit to levels comparable to the WT (Fig. 4*D*). However, the WT_F1307A disruption of the LIR motif did not influence the total expression of the GluN2B subunit when compared to the WT. These results indicate that the LIR domain positively regulates the lysosomal clearance of aberrant GluN2B subunits, thus preventing their accumulation. We then analyzed the surface expression *via* surface biotinylation assays to determine whether the accumulation of the R519Q subunits by LIR motif disruption was able to restore their surface expression. In accordance with our previous results, the surface expression of the R519Q_F1307A variant displayed equivalent surface expression to the R519Q variant alone, indicating that the trafficking defects of the R519Q variant cannot be rescued by disrupting the LIR domain alone. In addition, the WT_F1307A did not reduce the surface level compared to WT (Fig. 4*E*).

To further evaluate the role of the LIR motif in the autophagy-dependent degradation of the GluN2B subunits, we expressed the WT and R519Q LIR variants in ATG7 KO cells. Immunoblot analysis revealed similar expressions of all GluN2B subunits (Fig. 4*F*), further supporting that autophagy is essential in the removal of the R519Q variant. However, when we tested whether the disruption of the LIR motif would influence the surface expression in the ATG7 KO line, we discovered that the R519Q_F1307A variant was expressed on the surface at equal levels to the R519Q variant (Fig. 4*G*). These findings indicate that the LIR motif in the GluN2B subunit is crucial for the autophagic clearance of misfolded proteins but does not significantly alter receptor surface trafficking.

### Disruption of the LIR motif attenuates the lysosomal degradation of the R519Q DAV

**Figure 5:**
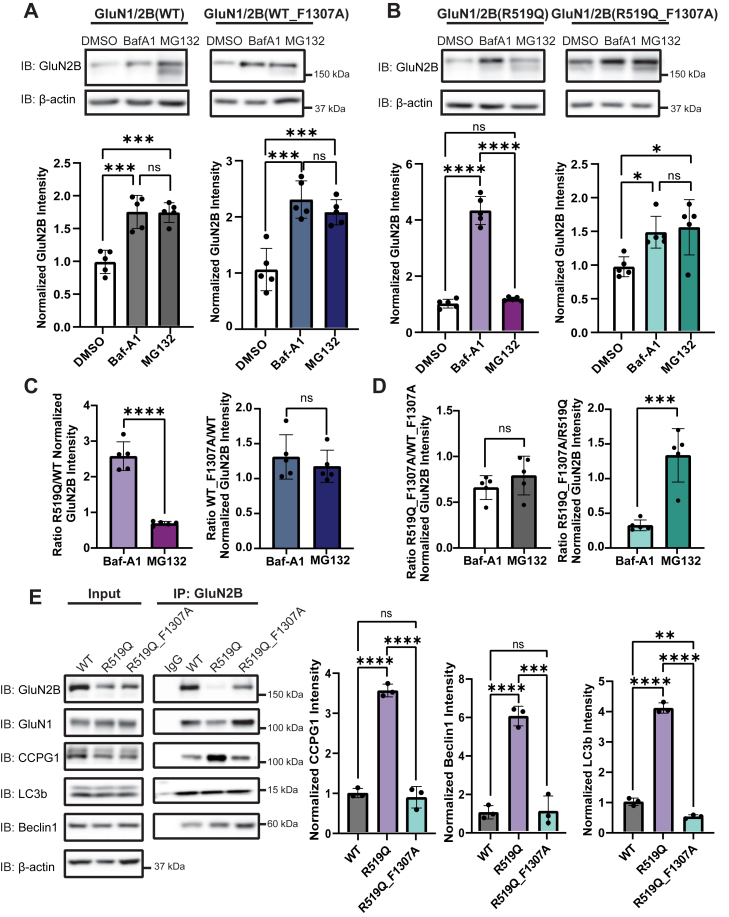
**Effects of LIR motif disruption on GluN2B autophagic degradation**. *A*, inhibition of the proteasome with MG132 (10 μM) and the lysosome with Baf-A1 (1 μM) for 6 h effect on the WT (*left*) and WT_F1307A (*right*) GluN2B subunit in HEK293T cells 48 h after transient transfection. β-actin served as the soluble total protein loading control (n = 5). *B*, inhibition of the proteasome with MG132 (10 μM) and the lysosome with Baf-A1 (1 μM) for 6 h effect on the R519Q (*left*) and the R519Q_F1307A (*right*) GluN2B subunit in HEK293T cells 48 h after transient transfection. β-actin served as the soluble total protein loading control (n = 5). *C*, ratio of the normalized accumulation of R519Q/WT GluN2B (*left*) and the ratio of the normalized accumulation of WT_F1307A/WT GluN2B (*right*) upon treatment with Baf-A1 and MG132. *D*, ratio of the normalized accumulation of R519Q_F1307A/WT_F1307A GluN2B (*left*) and the ratio of the normalized accumulation of R519Q_F1307A/R519Q GluN2B (*right*) upon treatment with Baf-A1 and MG132. *E*, co-immunoprecipitation of HEK293T cells exogenously expressing WT, R519Q, or R519Q_F1307A NMDARs with GluN2B variants was done to measure interaction with key autophagy proteins. Cells were treated with Baf-A1 (20 nM for 24 h), 24 h post transient transfection to enrich autophagy proteins for interaction. Quantification of the ratio of the protein of interest to GluN2B post co-IP is shown in the right panel (n = 3). All data are normalized to the appropriate loading control, and data are presented as mean ± SD. Statistical significance was determined using an unpaired two-tailed Student’s *t* test between two groups or an analysis of variance (ANOVA) followed by a *post hoc* Tukey test for comparison in multiple groups. Significance level defined as ns, not significant, ∗*p* < 0.05, ∗∗*p* < 0.01, ∗∗∗*p* < 0.001, ∗∗∗∗*p* < 0.0001.

To examine whether disruption of the LIR motif influences the sensitivity of the GluN2B subunit to degradation, we inhibited the lysosome and proteasome using Baf-A1 (1 μM, 6 h) and MG132 (10 μM, 6 h) respectively in HEK293T cells co-expressing GluN1 with either WT, WT_F1037A, R519Q, or R519Q_F1307A GluN2B subunits. In the case of WT GluN2B and WT_F1307A LIR disruption, there was equal accumulation with inhibition of either the lysosome or the proteasome, with a 1.7-fold accumulation in the WT upon inhibition of either pathway and a similar 2-fold increase in the WT_F1307A (Fig. 5*A*). Assessment of the ratios of GluN2B in response to proteolytic inhibition shows no significant change between the WT_F1307A LIR disruption and the WT GluN2B subunits (Fig. 5*C*). In contrast, the R519Q_F1307A variant exhibited a 1.6-fold increase in response to lysosomal inhibition and a 1.6-fold increase upon proteasomal inhibition. This contrasts with the R519Q variant that exhibits a 4.4-fold accumulation upon inhibition of the lysosome but no significant accumulation upon inhibition of the proteasome (Fig. 5*B*). Quantitative analysis determined that the R519Q_F1307A exhibited a 60% reduction in accumulation in response to Baf-A1 treatment but displays a 39% increase in accumulation of the GluN2B upon treatment with MG132 when compared to the R519Q variant. However, there was no change in the R519Q_F1307A GluN2B accumulation upon Baf-A1 or MG132 treatment when compared to the WT_F1307A GluN2B (Fig. 5*D*). These results demonstrate a shift towards proteasomal degradation when the LIR domain of the R519Q variant is disrupted, which resembles the degradation patterns observed in the WT_F1307A (Fig. 5, *A* and *D*).

Finally, we examined whether disruption of the LIR motif compromises interactions between GluN2B and proteins critical for autophagic degradation. Co-immunoprecipitation assays were performed using a GluN2B antibody to pull down HEK293T cell lysates overexpressing WT, R519Q, or R519Q_F1307A GluN2B subunits. Cells were treated with 20 nM Baf-A1 for 24 h to enrich autophagy proteins and enhance the detection of potential interactions. Probing for key autophagy proteins Beclin 1, LC3b, and CCPG1 revealed notable differences in their interaction profiles (Fig. 5*E*). GluN1 served as a positive control for interaction. Notably, while the R519Q_F1307A was able to interact with CCPG1, the interaction was substantially decreased compared to the R519Q variant, indicating that the LIR domain may strengthen interaction with the ER-phagy receptor. Furthermore, the R519Q variant showed a selective interaction with lipidated LC3b (LC3b-II) at a significantly greater ratio than the WT and R519Q_F1307A GluN2B subunit. Additionally, interactions with Beclin 1 followed a similar trend, in which the R519Q_F1307A greatly reduced the observed interaction. These results suggest that the presence of the R519Q variant promotes the interaction with autophagy proteins. Further, the LIR motif may enhance and facilitate the key interactions implicated in the autophagic degradation of the R519Q variant.

## Discussion

NMDARs are essential heterotetrametric ion channels comprising obligatory GluN1 and glutamate-binding GluN2 subunits that assemble within ER sheets, where precise folding and function of the ligand-binding domain is critical for stability, trafficking, and synaptic function (14, 15, 16). DAVs in the GluN2B subunit are highly concentrated in the LBD, demonstrating surface trafficking deficits almost exclusively to this domain (18, 50, 51). Furthermore, it has been demonstrated that variant distance from the bound ligand correlates directly with pathogenic severity (52). ER retention signals in GluN1/GluN2 subunits further enforce quality control, preventing premature trafficking of unassembled receptors (53, 54, 55, 56, 57, 58). While canonical ERAD handles minor folding errors, severe LBD misfolding, as exemplified by the R519Q variant, overwhelms this capacity, driving retention and necessitating alternative clearance mechanisms. Our study identifies CCPG1-mediated ER-phagy, acting in concert with a conserved GluN2B LIR motif, as this selective autophagic pathway that maintains proteostasis equilibrium while highlighting therapeutic opportunities to modulate variant clearance (18, 26, 59, 60).

**Figure 6:**
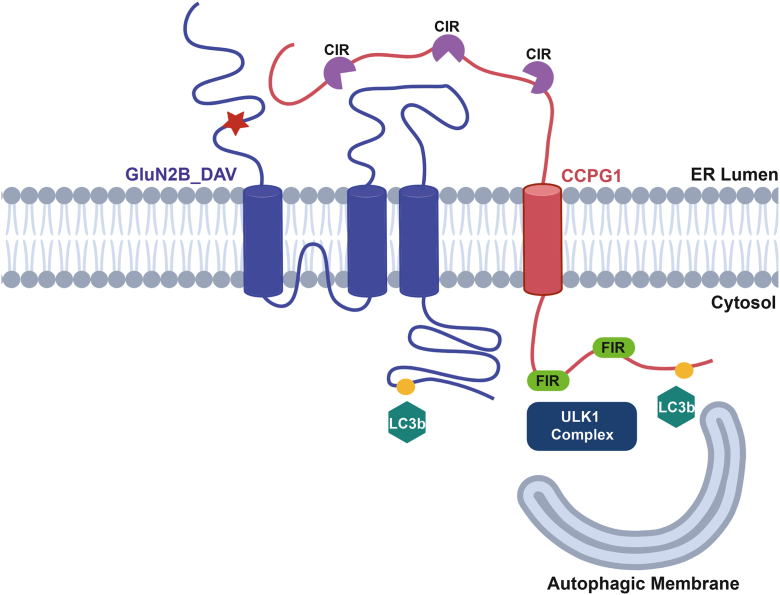
**Proposed mechanism of autophagic clearance of the GluN2B R519Q variant**. The GluN2B R519Q variant fails quality control measures in the ER, resulting in its retention within the ER lumen. CCPG1, shown in *red*, present in the ER sheets, can recognize the misfolded and accumulating R519Q variant, *blue*, and targets it for autophagic degradation *via* recruitment of autophagy machinery *via* interactions with the LIR, shown as *yellow circles*, and FIR domains, shown in *neon green*, of CCPG1. It is also possible that the LIR domain present on the cytoplasmic CTD of the GluN2B subunit is able to interact with autophagy machinery directly.

Our study elucidates the pathogenic influence of the R519Q variant in disrupting the protein homeostasis of the GluN2B subunit by promoting its clearance *via* the autophagy-lysosomal pathway. We report that (1) the R519Q variant significantly reduced the total and surface expression of the GluN2B subunit, resulting from subunit destabilization and retention within the ER due to impaired folding; (2) inhibition of autophagy by pharmacological inhibition, genetic knockout (ATG7), and siRNA-mediated knockdown of autophagy components identifies the R519Q variant as a preferential substrate for autophagy-lysosomal degradation; (3) misfolded R519Q subunits that fail quality control measures and are retained within the ER are selectively targeted to lysosomal degradation through key interactions with the ER-phagy receptor CCPG1 (Fig. 6); (4) we identify a novel and highly conserved LC3-interacting motif within the C-terminal domain of the GluN2B subunit; and (5) demonstrate this LIR motif influences the degradation of the GluN2B subunit and mediates interactions with key autophagy proteins. Collectively, these findings highlight a mechanistic pathway, in which the LIR-dependent engagement of ER-phagy receptors facilitates the selective autophagic clearance of misfolded GluN2B, providing new insight into the molecular quality control process that underlies *GRIN2B*-associated neurodevelopmental disorders.

The ubiquitin-proteasome system (ERAD) and the autophagy-lysosome pathway function in complementary union to maintain NMDAR proteostasis, with WT GluN2B subunit exhibiting balanced degradation as evidenced by comparable 1.7-2-fold accumulation upon Baf-A1 or MG132 treatment (Fig. 5*A*). Under physiological conditions, NMDAR proteostasis is maintained through ERAD, employing E3 ligases including NEDD4 (61), Mib2 (62), FBXO2 (63), and KTD13 (64), which have been implicated in the ubiquitination and retrotranslocation of NMDAR subunits. It has additionally been demonstrated that NMDARs are able to recruit the proteasome to the dendritic spine for degradation of synaptic proteins, and more recently, UBQLN4 has been shown to interact with GluN2B at synaptic sites and mediate its degradation (65, 66).

By contrast, autophagy clears misfolded substrates by ER-phagy, selective autophagy, and by endolysosomal degradation of internalized surface receptors. Notably, it should be considered that NMDAR-dependent autophagy induction during long-term depression, in which NMDAR-mediated calcium signaling can influence the mTOR pathway, further drives endosomal receptor turnover of AMPA receptors (67, 68, 69). Moreover, it is presumed that, similar to AMPA receptors, the endocytosis of NMDARs can result in lysosomal degradation *via* fusion with the endosome. The GluN2B LIR motif may regulate this surface-endosome-autophagy axis and recruit autophagic machinery to the synapse, warranting further investigation. Pathological R519Q misfolding disrupts the equilibrium between the proteolytic pathways, exceeding ERAD capacity and driving preferential autophagic clearance (4.4-fold Baf-A1 accumulation (Fig. 2*A*)), and routing cargo toward ER-phagy for lysosomal clearance of these ERAD-resistant substrates.

Consistent with established ER-to-lysosome associated degradation (ERALD) mechanisms (39), as seen with ATZ polymers and mutant procollagen undergoing calnexin-dependent FAM134b clearance (70), the R519Q GluN2B engages CCPG1, the predominant ER-phagy receptor in the ER sheets in our system. CCPG1 knockdown causes ∼6-fold R519Q accumulation while overexpression reciprocally decreases levels (Fig. 3, *C* and *D*), with enhanced co-IP interactions relative to WT or LIR-disrupted variants (Figs. 3*B*, 5*E*). CCPG1 is the only ER-phagy receptor with identified cargo-interacting regions (CIRs) within the ER lumen that directly bind misfolded ER clients. Furthermore, activation of the unfolded protein response (UPR) upregulates its expression, which would ideally position it to recognize R519Q variants at secretory ER sheets (71). Future investigations into the mechanisms of the CCPG1-GluN2B cargo recognition would identify if subunit misfolding results in ER stress, activates the UPR, and chaperone proteins that assist in NMDAR folding and assembly that may promote these interactions.

CCPG1 specifically contrasts marginal SEC62/FAM134b interaction (Fig. 3*B*), likely reflecting ER subdomain matching: NMDAR biogenesis occurs in CCPG1-enriched sheets rather than FAM134b-curved regions or SEC62 domains, suggesting that the subcellular ER distribution of GluN2B within the ER or the topological features of the ER membrane favor engagement by CCPG1. RTN3L provides modest tubular ER support (∼2-fold accumulation of R519Q upon RTN3L knockdown (Fig. S4*D*)) with non-preferential WT/R519Q binding (Fig. S4*C*), consistent with its roles in ER fragmentation under ER stress conditions, coordination of endosomal maturation, and unconventional protein secretion of misfolded proteins (72, 73, 74). Together, our findings refine the framework of receptor-specific ER-phagy in regulating GluN2B proteostasis: CCPG1 serves as the primary mediator of R519Q clearance from the ER, while RTN3L potentially facilitates alternative processing under ER stress conditions. While there is some specificity for ER-phagy substrates, there is also built-in redundancy among ER-phagy receptors. Whether the substantial accumulation of R519Q GluN2B upon CCPG1 knockdown is influenced by the combined perturbations of other ER-phagy receptors remains unresolved and warrants further investigation. Elucidating these relationships will be important for refining the degradation pathways that target pathogenic NMDAR variants.

We identified a novel, highly conserved F-type LIR motif (DTFVDL, residues 1305–1310) with the CTD of GluN2B, which is absent in other GluN subunits (Fig. 4*A-C*). Alanine substitution of the critical phenylalanine (F1307A) restores R519Q total expression to WT levels (Fig. 4*D*), phenocopying autophagy inhibition (Fig. 2*B*), while WT_F1307A remains unaffected, confirming LIR-mediated clearance specifically targets misfolded subunits. LIR disruption in R519Q reduces its autophagic clearance and shifts degradation towards the less efficient proteasomal degradation pathway as a fallback (60% reduced Baf-A1 accumulation, and 39% increased MG132 response (Fig. 5*B*, 5*D*)). Critically, LIR disruption fails to rescue R519Q surface trafficking (Fig. 4*E*, 4*G*), indicating persistent folding/assembly defects independent of degradation and subunit availability.

Our data support a model in which multivalent interactions with LC3b are mediated both by the LIR within GluN2B and by the ER-phagy receptor CCPG1 (Fig. 6). We propose a cooperative two-step model for GluN2B degradation by ER-phagy: (1) CCPG1 initially binds misfolded GluN2B within the ER lumen, potentially through its known cargo-interacting regions or by unidentified sequence; (2) LIR-mediated GluN2B LC3b engagement, stabilizing the CCPG1-GluN2B-LC3b complex. Together, multivalent interactions enhance recruitment, specificity, and efficiency beyond what either interaction could achieve alone. In this framework, CCPG1 and the GluN2B LIR cooperate to generate a multivalent assembly whose overall avidity increases as LC3b density rises on maturing phagophores, thereby strengthening cargo recruitment. Such multivalency may represent a general cargo recruitment mechanism to strengthen LC3b interactions to promote efficient autophagic flux (75). Within this model, the R519Q variant may increase CCPG1 engagement during the initial recognition step, while disruption of the R519Q LIR selectively impairs the stabilization phase. This layered requirement ensures that only pathologically misfolded subunits are selectively cleared while sparing WT receptors with intact LIRs but insufficient misfolding signal. Collectively, our findings suggest that the LIR in R519Q acts as a critical determinant for directing pathogenic GluN2B variants to autophagic degradation.

Our prior work demonstrated that BiX, a BiP activator, modestly rescues GluN2A variant trafficking by remodeling ER proteostasis (76). However, R519Q’s profound ER-phagy dependence suggests distinct strategies: rather than promoting folding/assembly, therapeutic modulation of CCPG1-LIR interaction could selectively clear variants while preserving WT homeostasis. This LIR-targeted approach may complement chaperone therapies for trafficking deficient NMDAR DAVs, particularly given GluN2B DAV diversity requiring functional classification for precision therapeutics (77). Proteomic studies mapping NMDAR-specific ER chaperones remain essential to identify synergistic proteostasis regulators.

## Conclusion

In summary, this study demonstrates that the pathogenic R519Q variant in GluN2B’s ligand-binding domain severely perturbs NMDAR proteostasis by reducing subunit stability, total/surface expression, and promoting ER retention, resulting in loss-of-function receptor. We establish that R519Q is preferentially cleared *via* the ER-phagy receptor CCPG1 and a novel, highly conserved LIR motif (DTFVDL, residues 1305–1310) in the CTD of GluN2B, which cooperatively engages LC3b to ensure selective autophagic degradation while sparing WT subunits. Critically, our results emphasize that the GluN2B R519Q subunit is subject to rigorous ER quality control measures—unrelieved by autophagy inhibition and rescue of the R519Q subunit pool-prevents surface rescue of the misfolded R519Q, highlighting early biogenesis as the primary site of pathogenicity for LBD disease-associated variants. These findings provide the first mechanistic insight into NMDAR clearance *via* ER-phagy under physiological and pathological conditions, offering a framework for understanding other *GRIN2B* missense and truncating mutations, and identifying CCPG1-LIR interactions as potential therapeutic targets to restore proteostasis in *GRIN* disorders.

### Limitations of the study

While our findings establish a compelling ER-phagy mechanism for R519Q GluN2B clearance, several limitations warrant consideration. First, experiments were conducted in HEK293T cells, which lack the neuronal context where NMDARs mediate synaptic function; validation in iPSC-derived neurons or primary cultures from *GRIN2B* variants will be essential to confirm physiological relevance. Second, GluN2B subunits were exogenously expressed using homozygous exogenous expression of variant subunits, while typically many patients present as heterozygous for these *de novo* variants. Further investigations should clarify whether the pathogenic effects of the R519Q variant have a dominant negative effect on the WT subunit. Third, we focused on a single exemplary variant (R519Q) despite screening multiple LBD DAVs; systematic analysis across a variant panel is needed to determine pathway generality. Fourth, while CCPG1 predominates, the modest RTN3L contribution and potential receptor redundancy remain unresolved, requiring combined knockdowns for quantitative dissection. Importantly, CCPG1 is ubiquitously expressed but is particularly enriched in high secretory tissues such as the pancreas, liver, kidney, thyroid and pituitary glands (78). Thus, the contribution of other ER-phagy receptors may be underrepresented in our exogenous model system and warrants further investigation. Finally, our study elucidates degradation mechanisms but does not address whether modulating CCPG1-LIR engagement can therapeutically rescue surface NMDAR function without off-target effects. These limitations highlight critical next steps, including neuronal models, multi-variant profiling, and functional rescue experiments, to translate these insights toward *GRIN* disorder therapeutics.

## Experimental procedures

### Reagents

The cycloheximide (#ALX-380–269) was purchased from Enzo Life Sciences. The (+)-MK801 maleate (#HB0004) was obtained from HelloBio. MG132 (#A2585) and sulfo-NHS-SS-biotin (#A8005) were purchased from APExBio. EDTA-free protease inhibitor cocktail (#04693159001) was purchased from Roche. TransIT-2020 Transfection Reagent (#MIR5400) was obtained from Mirus Bio and the HiPerFect transfection reagent (#301707) was obtained from Qiagen. Bovine serum albumin (BSA) (#A-421–25), G418 (G-418–10), and n-Dodecyl-β-maltoside (DDM) (#DDM5) were purchased from GoldBio. The 3-methyladenine (3-MA) (#HY-19312), SMER28 (#HY-100200), rapamycin (#HY-10219), and DAPI dihydrochloride (HY-D0814) were obtained from MedChemExpress. The bafilomycin-A1 (#11038) was obtained from Cayman Chemicals. Poly-L-lysine (#150177) was purchased from MP Biomedicals. All other chemicals were purchased from Sigma unless otherwise noted.

### Antibodies

The following primary antibodies were purchased and validated by the respective manufacturers. In addition, overexpression or knockdown experiments were used to validate the specificity of certain antibodies. The rabbit monoclonal anti-GluN2B antibody (#ab183942, RRID:AB_2889878, 1:3000), rabbit polyclonal anti-GluN2B (#ab73001, RRID:AB_1269571, 1:300), and rabbit monoclonal anti-Na^+^/K^+^ ATPase antibody (#ab76020, RRID:AB_1310695, 1:20,000;1:300) were purchased from Abcam. The mouse monoclonal anti-GluN2B (clone N59/20, #75–097, RRID:AB_10673405, 1:300) from NeuroMab was purchased from Antibodies Inc. The mouse monoclonal anti-β-actin (#A1978, RRID:AB_476692, 1:10,000) was purchased from Sigma Aldrich. The rabbit polyclonal anti-p62 (#PM045, RRID:AB_1279301,1:2000) was obtained from MBL. The rabbit monoclonal anti-CCPG1 (#80158s, RRID:AB_2935809, 1:2000), rabbit monoclonal anti-ATG7 (#8558s, RRID:AB_10831194, 1:1000), rabbit monoclonal anti-FAM134B (#83414s, RRID:AB_3696016, 1:2000) and rabbit monoclonal anti-Beclin 1 (#3495s, RRID:AB_1903911, 1:1000) came from Cell Signaling Technology. The rabbit polyclonal anti-LC3b (#NB100–2220, RRID:AB_10003146, 1:1500), the rabbit polyclonal anti-TEX264 (NBP1-89866, RRID:AB_11009420, 1:2000), and the rabbit polyclonal anti-SEC62 (NBP1-84045, RRID:AB_11037283, 1:2000) were obtained from Novus Biologicals. The rabbit polyclonal anti-calnexin (#ADI-SPA-860-F, RRID:AB_11178981, 1:500) was purchased from Enzo Life Sciences. The rabbit polyclonal anti-calnexin (#10427-2-AP, RRID:AB_2069033, 1:500), rabbit polyclonal anti-ATL3 (#16921-1-AP, RRID:AB_2290228, 1:2000) and rabbit polyclonal anti-RTN3 (#12055-2-AP, RRID:AB_2301357, 1:2000) were purchased from ProteinTech.

The following secondary antibodies were utilized for Western blot detection: HRP conjugated goat anti-mouse IgG (H + L) Superclonal Recombinant antibody (Invitrogen #A28177, RRID:AB_2536163, 1:10,000) and HRP conjugated goat anti-rabbit IgG (H + L) Superclonal Recombinant antibody (Invitrogen #A27036, RRID:AB_2536099, 1:10,000). Alexa Fluor secondary antibodies were obtained from Invitrogen used for confocal immunofluorescence are as follows: Alexa Fluor 488 goat anti-mouse antibody (#A11029, RRID:AB_2534088, 1:500), Alexa Fluor 488 goat anti-rabbit antibody (#A11034, RRID:AB_2758380, 1:500), Alexa Fluor 568 goat anti-mouse antibody (#A11031, RRID:AB_144696, 1:500), and Alexa Fluor 568 goat anti-rabbit antibody (#A11036, RRID:AB_10563566, 1:500).

### Plasmids and mutagenesis

The pcDNA3.1-*GRIN1* (OHu22255D, NM_007327, human) and the pcDNA3.1-*GRIN2B* (OHu26128D, NM_000834, human) and pcDNA3.1-*CCPG1* (OHu07897 C, NM_004748.5) were obtained from GenScript. The R519Q and F1307A mutations were introduced into the *GRIN2B* plasmid using QuikChange II site-directed mutagenesis kit (Agilent Genomics, #200523). All cDNA sequences were confirmed using DNA sequencing.

### Cell culture and transfection

HEK293T cells (ATCC, #CRL-3216 or Abgent, #CL1032) were maintained in Dulbecco’s Modified Eagle Medium (DMEM) (Cytiva #SH30243.01) containing 10% heat-inactivated fetal bovine serum (Cytiva, #SH30396.03HI) and 1% penicillin-streptomycin (Cytiva #SV30010) at 37°C in 5% CO_2_. Maintenance plates were passaged using 0.05% trypsin protease (Cytiva, #SH30236.01) upon reaching no greater than 90% confluency, and were used for experiments from passages 5 to 30. Cells were seeded in 10-cm dishes, 35-mm dishes or 6-well plates and grown until reaching 50 to 60% confluency. Cells were transiently transfected with a 1:1 ratio of GluN1:GluN2B plasmids using TransIT-2020 Transfection Reagent according to the manufacturer’s instructions. In order to prevent glutamate-mediated excitotoxicity, the media were supplemented with 50 μM (+)-MK-801 and 2.5 mM MgCl_2_ (Sigma, #208337). 48 h post-transfection, cells were harvested for protein analysis.

Stable HEK293T (Abgent, #CL1032) cell lines expressing NMDARs composed of GluN1_GluN2B and GluN1_GluN2B(R519Q) were generated using a G418 (Enzo Life Sciences, #ALX-380–013-G001) selection method in the presence of channel blockers MK-801 and MgCl_2_ as previously described. Briefly, cells were transfected with a 1:1 cDNA ratio of GluN1:GluN2B and GluN1:GluN2B(R519Q), respectively. Cells were selected using DMEM supplemented with 0.8 mg/ml G418 for 14 days. Cells were then maintained in DMEM supplemented with 0.4 mg/ml G418, and expression of GluN1 and GluN2B subunits were verified using Western blot analysis.

### Identification and pathogenic prediction of *GRIN2B* LBD missense variants

Pathogenicity predictions were obtained using Rhapsody (http://rhapsody.csb.pitt.edu/) *in silico* saturation mutagenesis for *GRIN2B* using the UniProt accession number Q13224, which evaluates the potential functional impact of variants based on protein sequence, structural context, and evolutionary conservation. The results were downloaded and recorded for comparative analysis. Mutations within the *GRIN2B* gene were downloaded from the ClinVar database and were filtered to include only those classified as single-nucleotide missense mutations. Variants that occur within the S1 and S2 segments of the LBD were defined according to Q13224 and relevant literature and used to isolate Rhapsody results for downstream analysis.

### Discovering the LIR motif and sequence alignment

To demonstrate the involvement of the LIR motif, the complete amino acid sequence of the *GRIN2B* gene was obtained by assessing the UniProt Database Q13224. A search was performed using the iLIR web server (https://ilir.warwick.ac.uk/) to identify peptide sequences associated with the LIR motif. Within the amino acid sequence of GluN2B, a canonical LIR fragment, DTFVDL, was identified at residue positions 1305 to 1310. Sequences of the *Homo sapiens* (human), *Mus musculus* (mice), *Rat norvegicus* (rat), *Canis lupus* *familiaris* (dog), and *Pan troglodytes* (chimpanzee) GluN2B subunits were obtained through Uniprot Database (respective accession Numbers: Q13224, Q01097, Q00960, Q5R1P3, H2Q5I0). Sequence alignment was performed using Clustal Omega.

### RNA interference (RNAi)-mediated gene knockdown

HEK293T cells stably expressing GluN1_GluN2B(R519Q) NMDARs were grown until they reached 60% confluency. 50 nM of siRNA was transfected using HiPerfect Transfection Reagent (Qiagen # 301717) according to the manufacturer’s instructions. 48 h after transfection, cells were harvested and subjected to SDS-PAGE and Western blot analysis. At least two distinct siRNAs were used for each gene knockdown, independently and in combination, in order to minimize off-target effects and increase knockdown efficiency. Non-targeting (NT) scrambled control pool siRNA was used as a negative control (Dharmacon #D-001810–01–20).

The following human ON-TARGET*plus* siRNA duplexes were obtained from Dharmacon: CCPG1-1 (J-013998–05–0005), CPPG1-2 (J-013998–06–0005), CCPG1-3 (J-013998–07–0005), CCPG1-4 (J-013998–08–0005), RTN3-1 (J-020088–09), RTN3-2 (J-020088–10), ATG7-1 (J-020112–05–0005), ATG7-2 (J-020112–06–0005), LC3a-1 (J-013579–07–0005), LC3a-2 (J-013579–08–0005), LC3b-1 (J-012846–05–0005), LC3b-2 (J-012846–08–0005). LC3c-1 (J-032399–09–0005), and LC3c-2 (J-032399–11–0005). The designation of GENE-1 and GENE-2 indicates two distinct siRNA sequences against each mRNA transcript.

### SDS-PAGE and Western blot

Cells were harvested using 4 °C Dulbecco’s phosphate-buffered saline (DPBS) (Corning #21–030-CVR) and lysed using lysis buffer (50 mM Tris-HCl, pH 7.4, 150 mM NaCl, 2 mM DDM supplemented with protease inhibitor cocktail (Roche, #04693159001). Lysates were cleared *via* centrifugation (20,000 × *g*, 10 min, at 4 °C) and the supernatant was collected as total protein. Protein concentrations were measured using the MicroBCA Protein Assay Kit (ThermoFisher, #23235). 30 μg of cell lysates were loaded with Laemmli buffer (Biorad #1610747) with 10% β-mercaptoethanol (βME) (Sigma #M6250). Samples were subjected to SDS-PAGE and separated on either an 8% or a 4 to 20% acrylamide gel. The Precision Plus Protein Kaleidoscope Prestained Protein Standard (Biorad #31610375) and/or the Broad Range Prestained Protein Marker (Proteintech #PL00002) were used as molecular weight markers. Western blot analysis was performed, and gels were transferred to 0.45 μm nitrocellulose membrane (Biorad #1620115) and then blocked in 5% skim milk in TBST buffer for 1 h at room temperature. Membranes were probed with the appropriate antibodies and dilutions listed above. β-actin and Na^+^/K^+^ ATPase were used as loading controls for total protein lysate and plasma membrane proteins respectively. Membrane visualization was performed using chemiluminescent substrates SuperSignal West Pico PLUS Chemiluminescent Substrate (ThermoFisher #34578) or SuperSignal West Femto Maximum Sensitivity Chemiluminescent Substrate (ThermoFisher #34096) and images were acquired using Azure 600 Imager (Azure Biosystems). Protein band intensity was quantified using ImageJ software from the NIH. Total protein was normalized to the loading control and then the experimental control (DMSO vehicle or WT).

### Biotinylation of cell surface proteins

HEK293T cells were plated on poly-L-lysine coated plates and cultured as described. Surface biotinylation experiments were performed according to published procedures (76, 79). Briefly, cells were transfected with the corresponding GluN1 and GluN2B plasmids for 48 h. If being treated with an autophagy regulator, cells were treated 24 h after transient transfection for 24 h. Intact cells were washed with ice-cold DPBS and incubated with 0.5 mg/ml sulfo-NHS SS Biotin (APExBio #A8005), a membrane impermeable biotinylation reagent, dissolved in DPBS containing 1 mM CaCl_2_ and 0.5 mM MgCl_2_ (DPBS + CM) for 30 min at 4 °C to label surface membrane proteins. Cells were then twice incubated with 50 mM glycine in DPBS + CM for 5 min at 4 °C to quench the labeling reaction. Sulfhydryl groups were blocked by incubating the cells with 5 nM N-ethylmaleimide (NEM) for 15 min at room temperature. Cells were scraped off in lysis buffer (50 mM Tris-HCl, pH 7.4, 150 mM NaCl, 2 mM DDM) supplemented with 5 mM NEM and protease inhibitor cocktail. Cells were solubilized overnight at 4 °C and lysates were cleared by centrifugation at (20,000 × *g*, 10 min at 4 °C). The concentration of the supernatant containing the biotinylated surface proteins was measured using a MicroBCA assay. Biotinylated surface proteins were affinity-purified by incubating the supernatant with 70 μl NeutrAvidin-conjugated agarose beads (ThermoFisher #29201) at 4 °C for 18 to 24 h. The samples were subjected to centrifugation (5000 × *g*, 1 min, at 4 °C). The beads were washed 6X with solubilization buffer (1X TBS, 1% Triton X-100). Surface proteins were eluted from beads by vortexing at room temperature for 20 min with 120 μl of elution buffer (2X Laemmli sample buffer, 100 mM DTT, 6M urea pH 6.8) followed by SDS-PAGE and Western blot analysis.

### Cycloheximide-chase assay

HEK293T cells stably expressing GluN1_GluN2B and GluN1_GluN2B(R519Q) NMDAR receptors were treated with 100 μg/ml cycloheximide to inhibit protein translation and synthesis. Cells were chased for the indicated amount of time, harvested, and lysed for SDS-PAGE and Western blot analysis to assess total protein.

### Confocal immunofluorescence staining and confocal microscopy

HEK293T cells were fixed with 4% paraformaldehyde in DPBS for 20 min and then blocked with 3% BSA in DPBS for 30 min. To investigate accumulation in the ER, membranes were permeabilized by incubating the fixed cells with 0.2% saponin for 20 min at room temperature prior to blocking. The cells were then incubated with 3% BSA containing mouse-monoclonal anti-GluN2B (NeuroMab #75–097, 1:300), rabbit polyclonal anti-Calnexin (1:500), and 0.2% saponin overnight at 4 °C. The cells were washed thoroughly with DPBS after primary staining and incubated with Alexa 488-conjugated goat anti-mouse antibody (1:500) and Alexa 568-conjugated goat anti-rabbit antibody (1:500) diluted in 3% BSA for 1 h at room temperature. Cells were permeabilized with 0.2% saponin and incubated with 1 μg/ml DAPI for 10 min. The coverslips were then mounted with Fluoromount-G (Invitrogen, #00–4958–02) and sealed. An Olympus IX-81 Fluoview FV3000 confocal laser scanning microscope was used. A 60X oil objective was used to collect high-resolution images using FV31s-SW software. The images were analyzed using ImageJ software.

### Immunoprecipitation

Cell lysates (1000 μg) were pre-cleared with 30 μl of Protein A/G plus-agarose beads (Santa Cruz Biotechnology, sc-2003) and 1 μg normal mouse IgG (Santa Cruz Biotechnology, #sc-2025) for 1 h at 4 °C to remove nonspecific binding proteins. The pre-cleared lysates were incubated with 2 μg of mouse anti-GluN2B antibody (NeuroMab #75–097) for 1 h at 4 °C, and then 50 μl of Protein A/G-plus agarose beads were added and incubated overnight at 4 °C. The beads were collected by centrifugation at (8000 × *g*,1 min) and washed 3X with lysis buffer (TBS, 1% Triton X-100). Protein complexes were eluted by incubating with 100 μl of Laemmli sample buffer with βME. The eluents were subjected to SDS-PAGE on a 4 to 20% gradient gel and Western blot analysis was performed. IgG serves as a negative control.

### Quantification and statistical analyses

Western blot bands were quantified using ImageJ. For each lane, the intensity of the protein of interest (POI) was normalized to the corresponding loading control (β-actin and Na^+^/K^+^ ATPase) to correct for lane-to-lane loading variability. For each biological replicate, the normalized values were then sum-normalized by dividing each lane’s value by the total signal for that biological replicate, ensuring that differences in overall signal intensity between replicates were minimized by placing all replicates on the same scale. To allow comparison across experiments, the mean value of the control condition across all replicates was calculated, and sum-normalized values were then divided by the mean control value, such that the control condition is set to 1 across the entire dataset. This final step converts all measurements into values relative to the control, enabling direct comparison of experimental conditions across replicates. All data were analyzed using GraphPad Prism software. All data are presented as mean ± SD. Statistical significance was evaluated between two groups using two-tailed Student’s *t* test or a Mann–Whitney *U* test for nonparametric data. Comparison between multiple groups, a one-way ANOVA followed by a *post hoc* Tukey or Dunnett’s *post hoc* test for comparisons. An assigned *p*-value < 0.05 was considered statistically significant. ∗, *p* < 0.05; ∗∗, *p* < 0.01; ∗∗∗, *p* < 0.001; ∗∗∗∗, *p* < 0.0001.

## Data availability

All data are contained within the manuscript. All original Western blot images are included in Fig. S5.

## Supporting information

This article contains supporting information.

## Conflict of interest

The authors declare that they have no conflicts of interest with the contents of this article.
