# Supplementary material for: A GluN2B disease-associated variant promotes the degradation of NMDA receptors via autophagy
Source: J Biol Chem. 2026 Jan 10;302(3):111147. doi: 10.1016/j.jbc.2026.111147 (PMC12882719; doi:10.1016/j.jbc.2026.111147)
Supplement: Supplementary Material [file mmc2.pdf]

Supporting information

A GluN2B disease-associated variant promotes the degradation of NMDA receptors via autophagy

Taylor M. Benske[1], Marnie P. Williams[1], Pei-Pei Zhang[1], Adrian J. Palumbo[1], Ting-Wei Mu[1*]

[1]Department of Physiology and Biophysics, Case Western Reserve University School of Medicine,

10900 Euclid Ave, Cleveland, Ohio 44106, USA.

*Corresponding author: Ting-Wei Mu

Mailing Address: Department of Physiology and Biophysics, Case Western Reserve University School of Medicine, 10900 Euclid Ave, Cleveland, OH 44106, USA

Email: tingwei.mu@case.edu

Phone: 216-368-0750

ORCID: 0000-0002-6419-9296

Running Title: Autophagy degrades an NMDA receptor variant

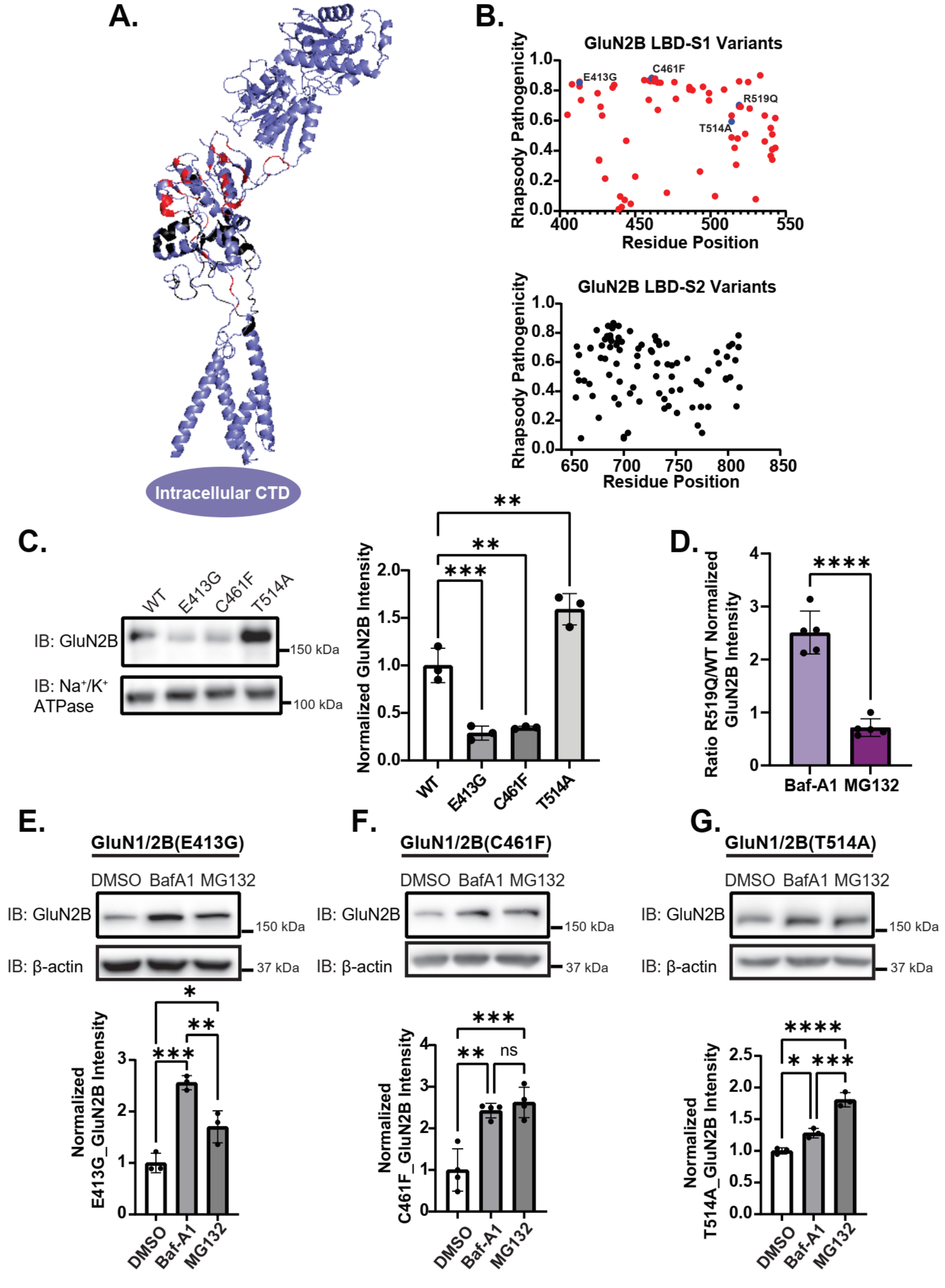

Supplemental Figure S1: Predicted pathogenicity and characterization of GluN2B LBD variants

(**A**) Structure of an isolated GluN2B subunit with known variants within the LBD-S1 colored in red and variants within the LBD-S2 colored in black (PDB:9ARI). (**B**) Rhapsody pathogenicity

predictions for variants within the GluN2B LBD graphed against residue position. LBD-S1 in red and LBD-S2 in black. DAVs investigated in this study are labeled and highlighted in blue. (**C**). Surface biotinylation assay to monitor the influence of LBD DAVs, E413G, C461F, and T514A on the surface expression of NMDARs 48 hrs post transient transfection. Na^+/K^+ ATPase served as a membrane protein loading control (n=4). **(D)** Ratio of the normalized accumulation of R519Q/WT GluN2B upon treatment with Baf-A1 and MG132 from experiments shown in Fig. 2A. (**E-G**) Effect of inhibition of the proteasome with MG132 (10 μM) or the lysosome with Bafilomycin-A1 (1 μM) for 6 hr on the GluN2B subunit in HEK293T cells expressing E413G (n=3) (**E**), C461F (n=4) (**F**), or T514A (n=3) (**G**) NMDARs. β-actin serves as the soluble total protein loading control. Data are presented as mean ± SD. Statistical significance was determined using an unpaired two-tailed Student's t-test between two groups or an analysis of variance (ANOVA) followed by a post-hoc Tukey test for comparison in multiple groups. Significance level defined as *p<0.05, **p<0.01, ***p<0.001, ****p<0.0001.

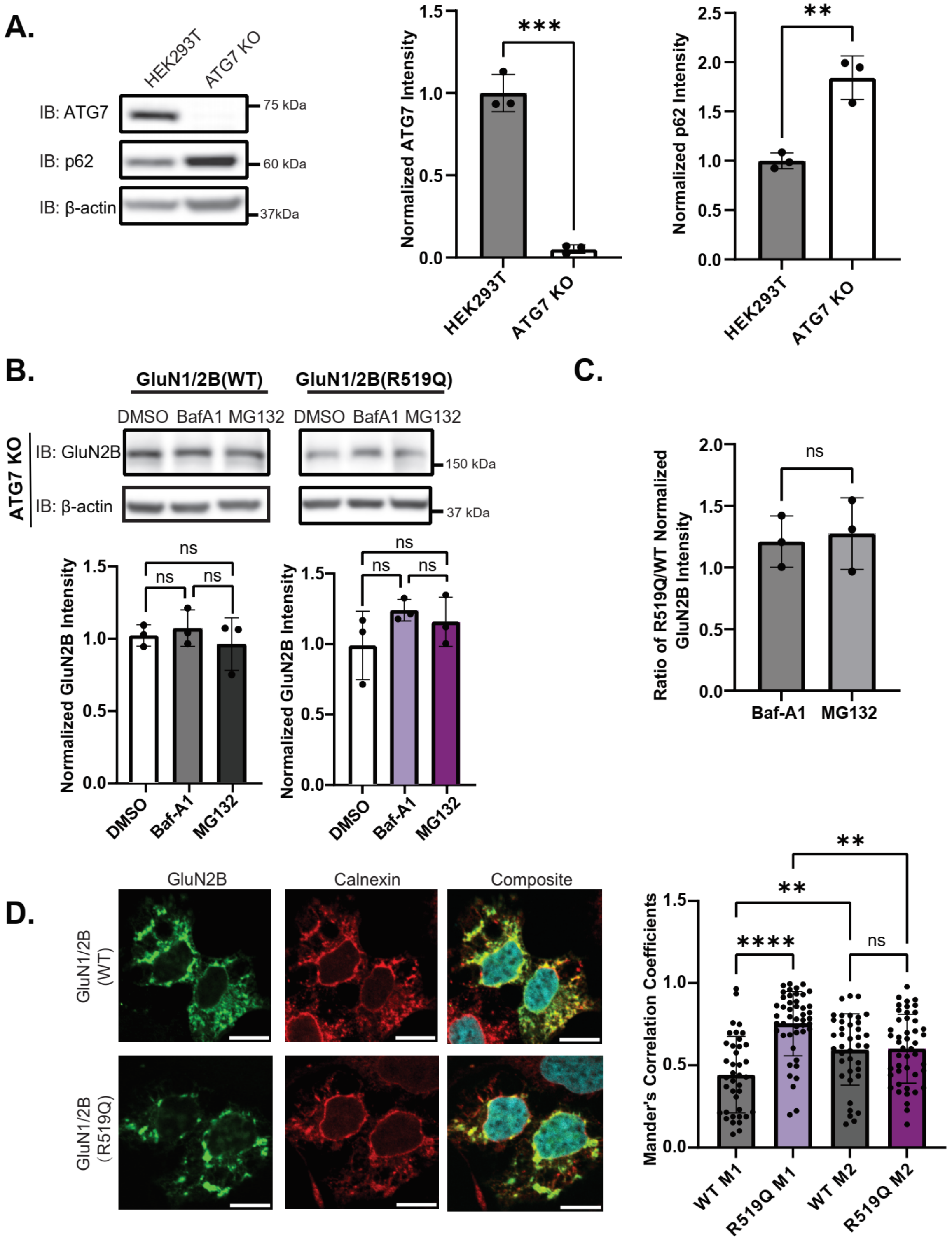

Supplemental Figure S2: Genetic depletion of autophagy in ATG7 KO lines results in ER retention of GluN2B without compensatory degradation

(A) Validation of HEK293T ATG7 KO line. Immunoblot was used to quantify levels of ATG7 in HEK293T and ATG7 KO lines. p62 was used to assess impaired autophagic flux. β-actin served

as the soluble total protein loading control (n=3). **(B)** Inhibition of the proteasome with MG132 (10 μM) and the lysosome with Baf-A1 (1 μM) for 6 hrs on the effect of the GluN2B subunit in ATG7 KO HEK293T cells expressing recombinant WT or R519Q NMDARs. β-actin served as the soluble total protein loading control (n=3). **(C)** Ratio of the normalized accumulation of R519Q/WT GluN2B upon treatment with Baf-A1 and MG132. **(D)** Immunofluorescence images of GluN2B (green) and Calnexin, an ER marker (red) to assess ER accumulation in ATG7 KO cells. Mander's coefficients are reported, where M1= ratio of GluN2B/Calnexin and M2= ratio of Calnexin/GluN2B (scale bar: 10 μm, n>30 cells) and statistical significance was determined using a Mann-Whitney test. All data are normalized to the appropriate loading control, and data are presented as mean ± SD. Statistical significance was determined using an unpaired two-tailed Student's t-test between two groups (**A, C**) or an analysis of variance (ANOVA) followed by a post-hoc Tukey test for comparison in multiple groups (**B**). Significance level defined as *p<0.05, **p<0.01, ***p<0.001, ****p<0.0001, ns, not significant.

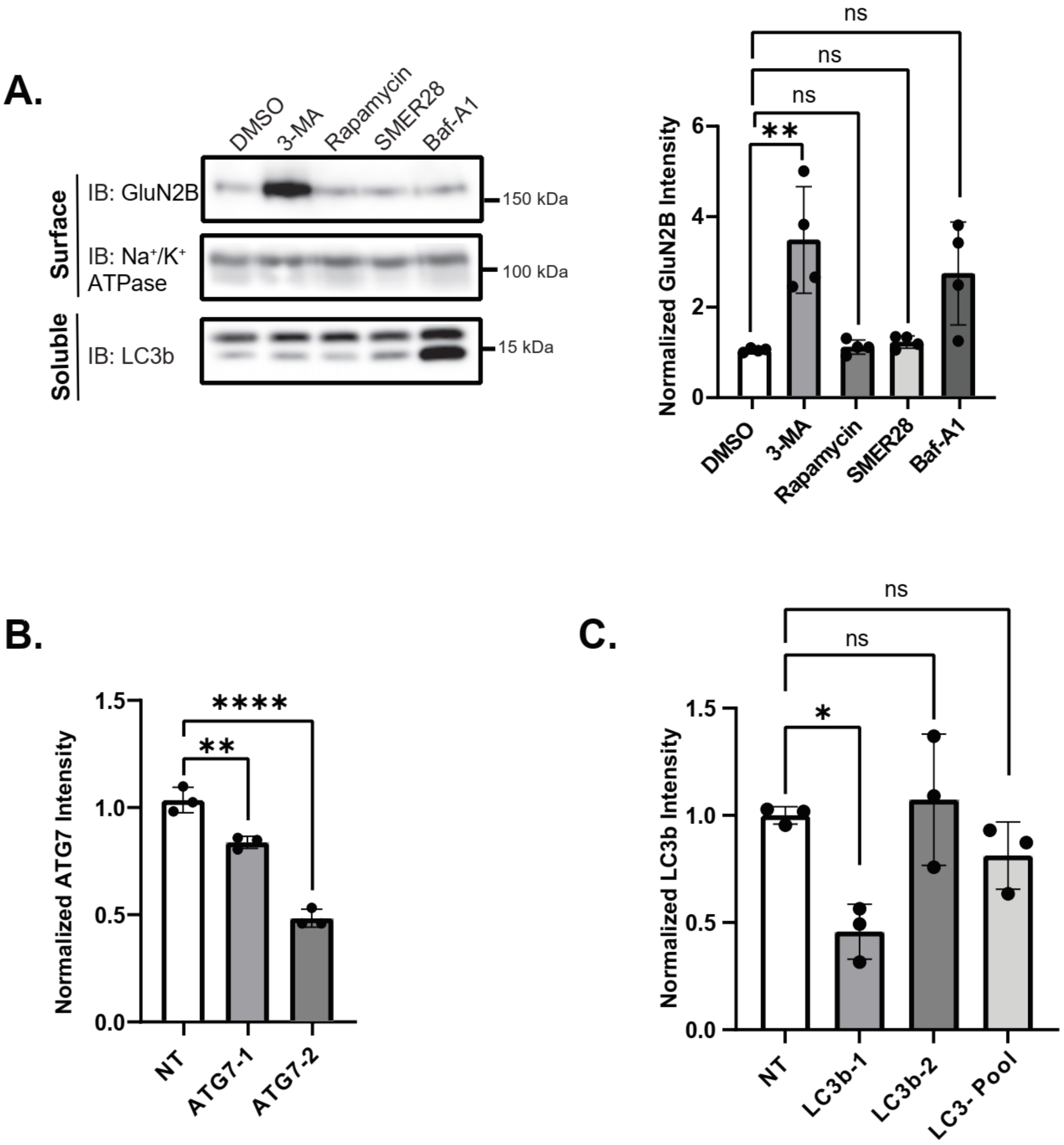

Supplemental Figure S3: Pharmacological modulation of autophagy on the surface expression R519Q GluN2B and validation of siRNA knockdown efficiency

(A) HEK293T cells stably expressing R519Q NMDARs were treated with autophagy activators (SMER28 10 µM, Rapamycin 100 nM) and inhibitors of autophagy (3-MA 50 mM and Baf-A1 20 nM) for 24 hrs. Surface biotinylation assays were performed to monitor the influence on the surface expression 24 hrs after drug treatment. Na^+/K^+ ATPase served as a membrane protein loading control (n=3). **(B)** Quantification of siRNA-mediated knockdown efficiency of ATG7 supporting experiments shown in Fig. 2F (n=3). (**C**) Quantification of siRNA-mediated knockdown efficiency of LC3b, supporting experiments shown in Fig. 2G (n=3). Data are presented as mean ± SD. Statistical significance was determined using an analysis of variance (ANOVA) followed by a post-hoc Tukey test for comparison in multiple groups. Significance level defined as **p<0.01, ****p<0.0001, ns, not significant.

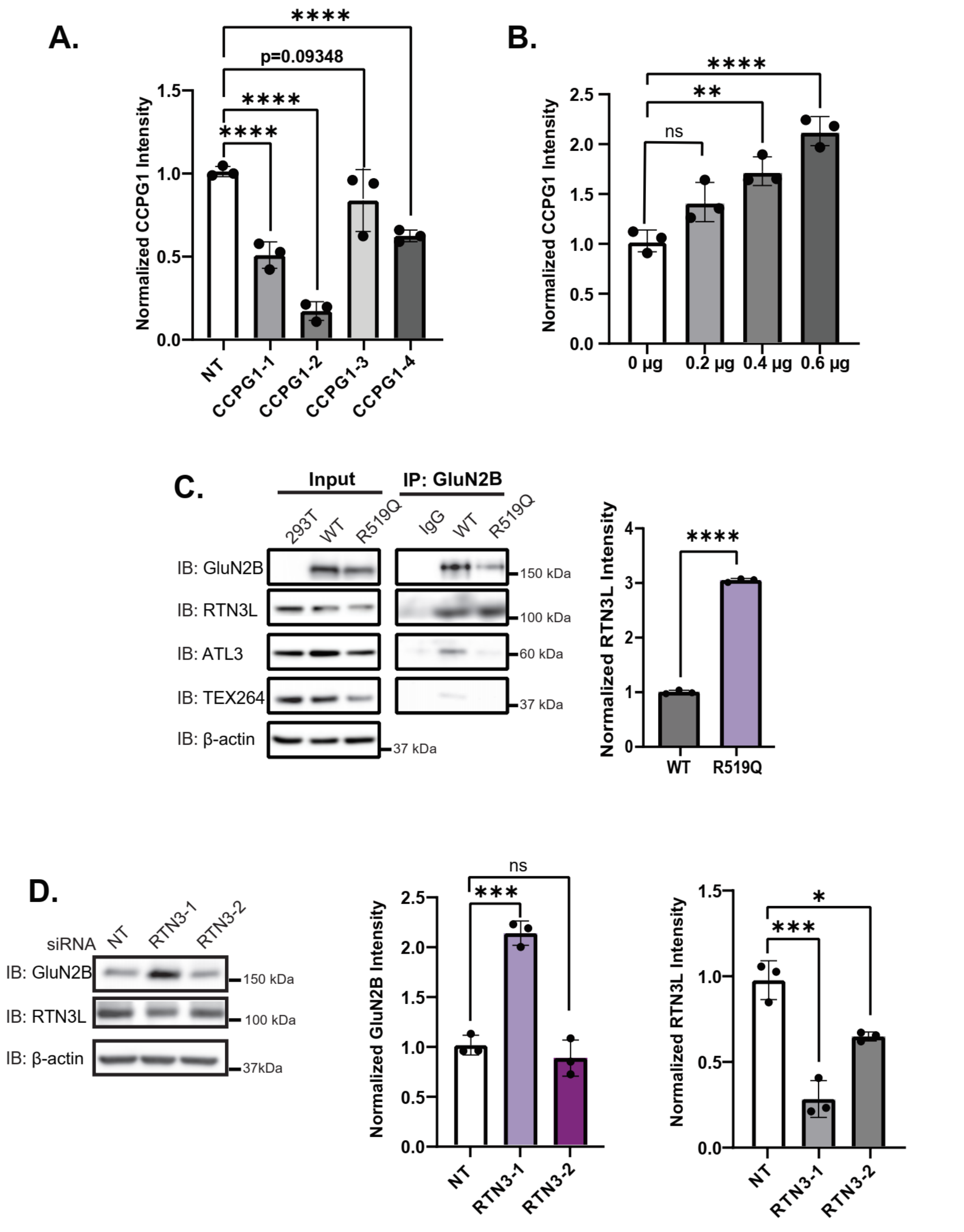

Supplemental Figure S4: Tubular ER-phagy receptor RTN3L interacts with R519Q GluN2B variants

(**A**) Quantification of siRNA-mediated knockdown efficiency of CCPG1 supporting experiments shown in Fig. 3C (n=4). (**B**) Quantification of overexpressed CCPG1 protein levels supporting

experiments shown in Fig. 3D (n=3). (**C**) Co-immunoprecipitation of HEK293T cells exogenously expressing R519Q GluN2B variants was done to measure interaction with tubular ER-phagy receptors RTN3L, ATL3, and TEX264. Cells were treated with Baf-A1 (20 nM for 24 hrs), 24 hrs post transient transfection to enrich autophagy proteins for interaction. Quantification of the ratio of the protein of interest to GluN2B post co-IP is shown in the right panel (n=3). (**D**) siRNA knockdown of RTN3L effects on GluN2B expression in HEK293T cells stably expressing R519Q NMDARs. Immunoblots performed 48 hrs after knockdown. Non-targeting (NT) siRNA was used as a control for each condition, and two independent siRNA constructs were used to knockdown gene expression. β-actin served as the soluble total protein loading control. Quantification of R519Q GluN2B protein levels and siRNA-mediated knockdown efficiency of RTN3L is shown in the right panels (n=3). All data are normalized to the appropriate loading control, and data are presented as mean ± SD. Statistical significance was determined using an unpaired two-tailed Student's t-test between two groups or an analysis of variance (ANOVA) followed by a post-hoc Tukey test for comparison in multiple groups. Significance level defined as *$p<0.05$, **$p<0.01$, ***$p<0.001$, ****$p<0.0001$, ns, not significant.

IB: GluN2B

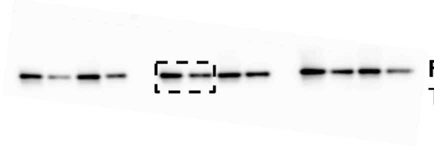

Figure 1C
Top panel

IB: β-actin

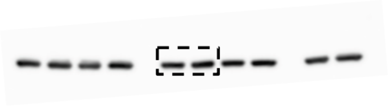

Figure 1C
Bottom panel

IB: GluN2B

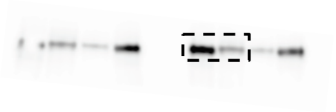

Figure 1D
Top panel

IB: Na^{+}/K^{+} ATPase

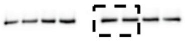

Figure 1D
Bottom panel

IB: GluN2B

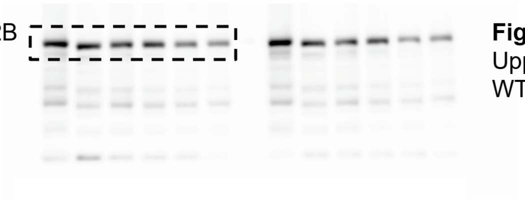

Figure 1E
Upper panel
WT CHX

IB: β-actin

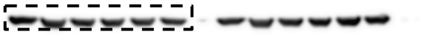

Figure 1E
Upper panel
WT CHX

IB: GluN2B

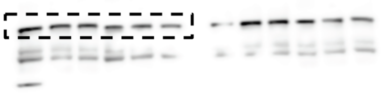

Figure 1E
Lower panel
R519Q CHX

IB: β-actin

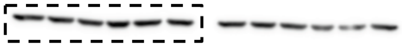

Figure 1E
Lower panel
R519Q CHX

IB: GluN2B

IB: β-actin

IB: GluN2B

IB: β-actin

IB: GluN2B

IB: β-actin

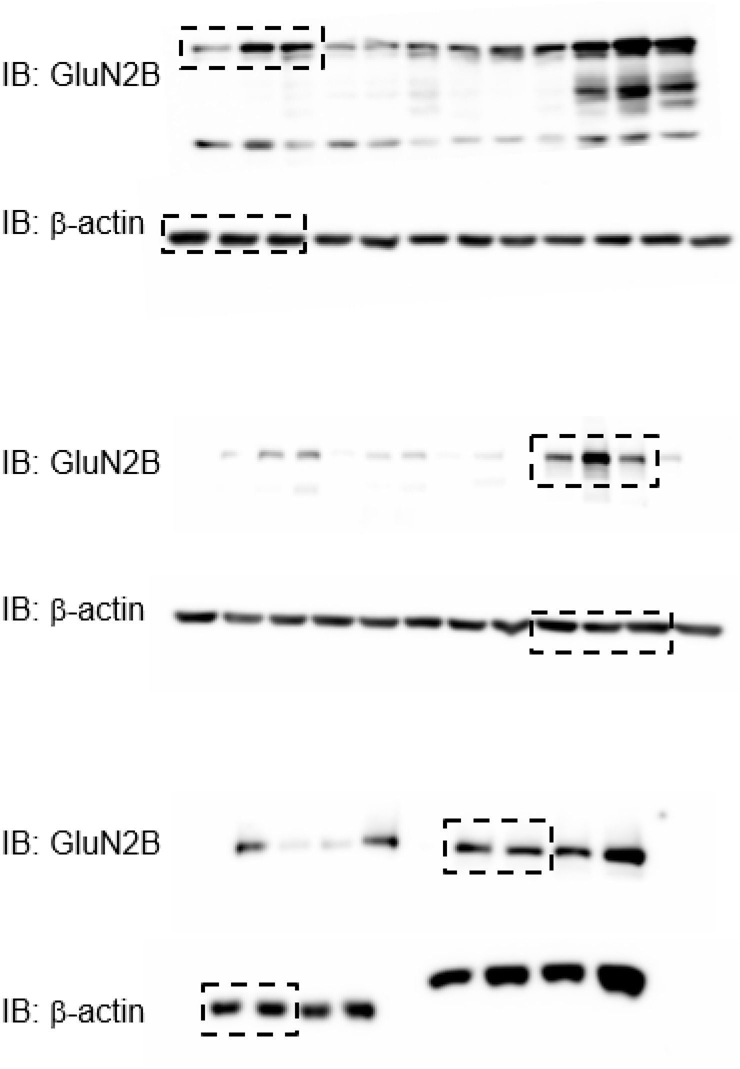

Figure 2A Left top panel

Figure 2A Left bottom panel

Figure 2A Right top panel

Figure 2A Right bottom panel

Figure 2B Top panel

Figure 2B Bottom panel

IB: GluN2B

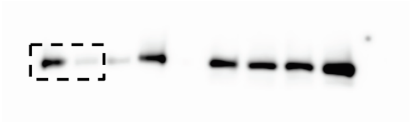

Figure 2C
Top panel

IB: Na+/K+ ATPase

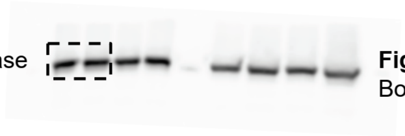

Figure 2C
Bottom panel

IB: GluN2B

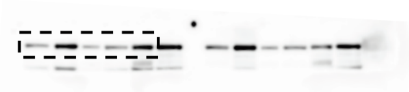

Figure 2E
Top panel

IB: p62

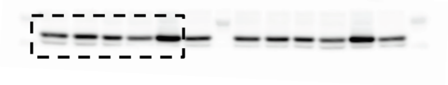

Figure 2E
Middle panel

IB: β-actin

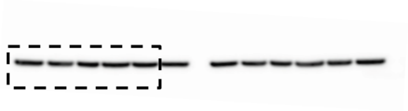

Figure 2E
Bottom panel

IB: GluN2B

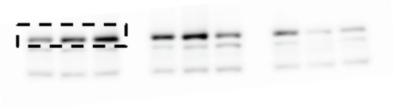

Figure 2F
Top panel

IB: ATG7

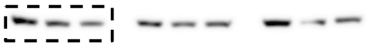

Figure 2F
Middle panel

IB: β-actin

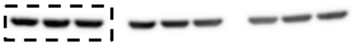

Figure 2F
Bottom panel

IB: GluN2B

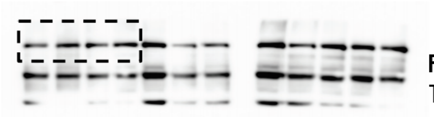

Figure 2G
Top panel

IB: LC3b

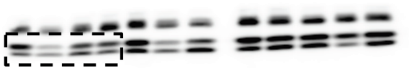

Figure 2G
Middle panel

IB: β-actin

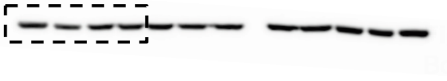

gure 2G
ottom panel

Figure 3B - Input

IB: GluN2B

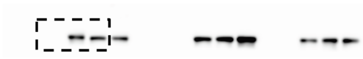

Figure 3B
Left top panel

IB: CCPG1

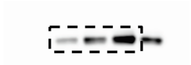

Figure 3B
Left 2nd panel

IB: FAM134b

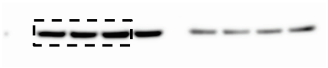

Figure 3B
Left 3rd panel

IB: SEC62

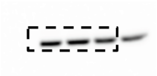

Figure 3B
Left 4th panel

IB: β-actin

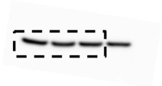

Figure 3B
Left bottom panel

Figure 3B – Pulldown (IP)

IB: GluN2B

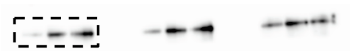

Figure 3B
Right top panel

IB: CCPG1

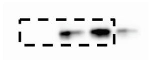

Figure 3B
Right 2nd panel

IB: FAM134b

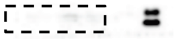

Figure 3B
Right 3rd panel

IB: SEC62

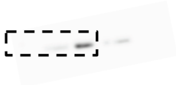

Figure 3B
Right bottom panel

IB: GluN2B

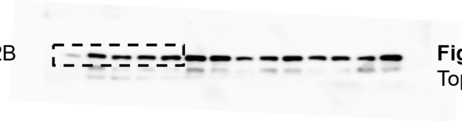

Figure 3C
Top panel

IB: CCPG1

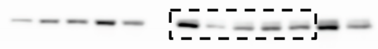

Figure 3C
Middle panel

IB: β-actin

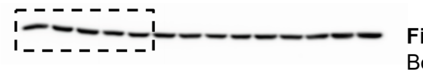

Figure 3C
Bottom panel

IB: GluN2B

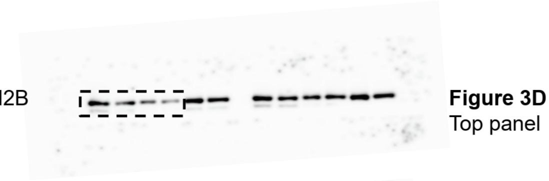

Figure 3D
Top panel

IB: CCPG1

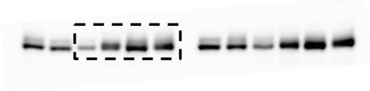

Figure 3D
Middle panel

IB: β-actin

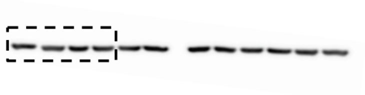

Figure 3D
Bottom panel

IB: GluN2B

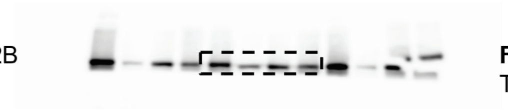

Figure 4D
Top panel

IB: β-actin

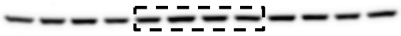

Figure 4D
Bottom panel

IB: GluN2B

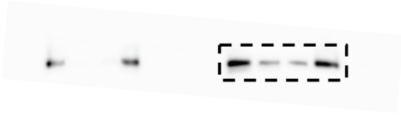

Figure 4E
Top panel

IB: Na^{+}/K^{+} ATPase

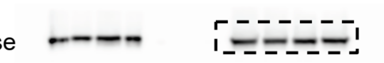

Figure 4E
Bottom panel

IB: GluN2B

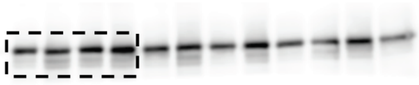

Figure 4F
Top panel

IB: β-actin

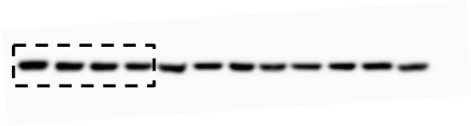

Figure 4F
Bottom panel

IB: GluN2B

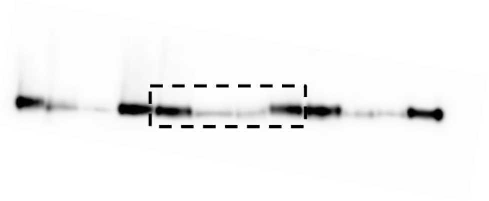

Figure 4G
Top panel

IB: Na^{+}/K^{+}
ATPase

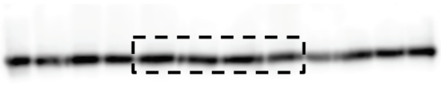

Figure 4G
Bottom panel

IB: GluN2B

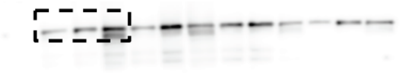

Figure 5A
Left top
panel

IB: β-actin

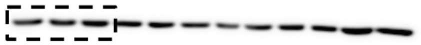

Figure 5A
Left bottom
panel

IB: GluN2B

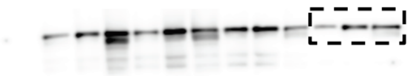

Figure 5A
Right top
panel

IB: β-actin

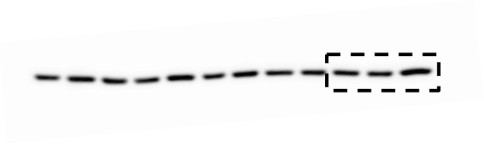

Figure 5A
Right bottom
panel

IB: GluN2B

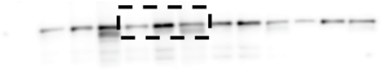

Figure 5B
Left top
panel

IB: β-actin

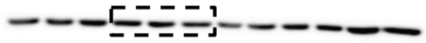

Figure 5B
Left bottom
panel

IB: GluN2B

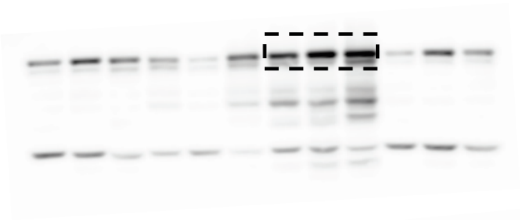

Figure 5B
Right top
panel

IB: β-actin

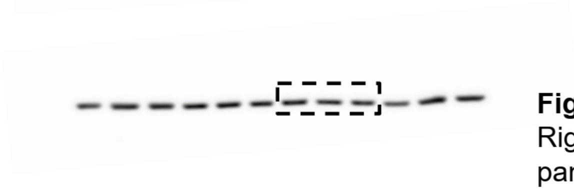

Figure 5B
Right bottom
panel

IB: GluN2B

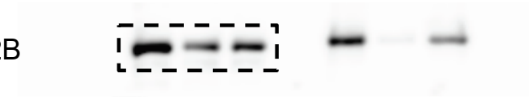

Figure 5E
Left top panel

IB: GluN1

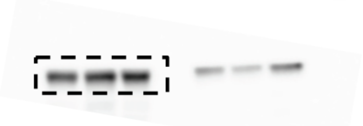

Figure 5E
Left 2nd panel

IB: CCPG1

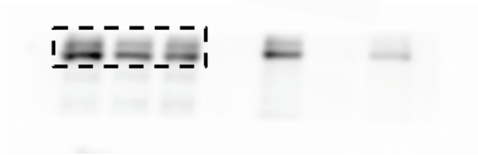

Figure 5E
Left 3rd panel

IB: LC3b

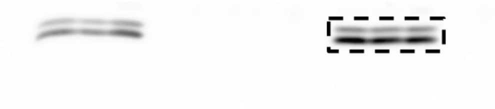

Figure 5E
Left 4th panel

IB: Beclin1

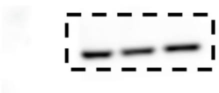

Figure 5E
Left 5th panel

IB: β-actin

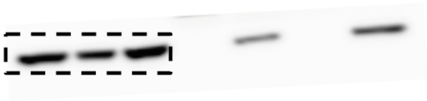

Figure 5E
Left bottom panel

Figure 5E – Pulldown (IP)

IB: GluN2B

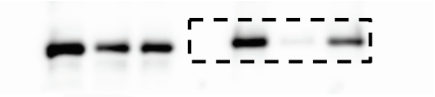

Figure 5E
Right top panel

IB: GluN1

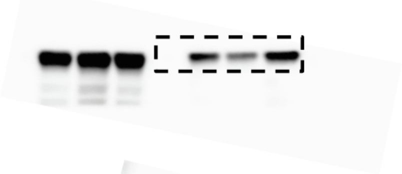

Figure 5E
Right 2nd panel

IB: CCPG1

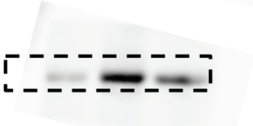

Figure 5E
Right 3rd panel

IB: LC3b

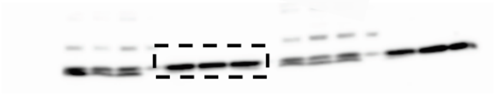

Figure 5E
Right 4th panel

IB: Beclin1

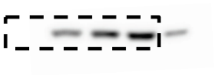

Figure 5E
Right bottom panel

Supplemental Figure 1C/E/F/G

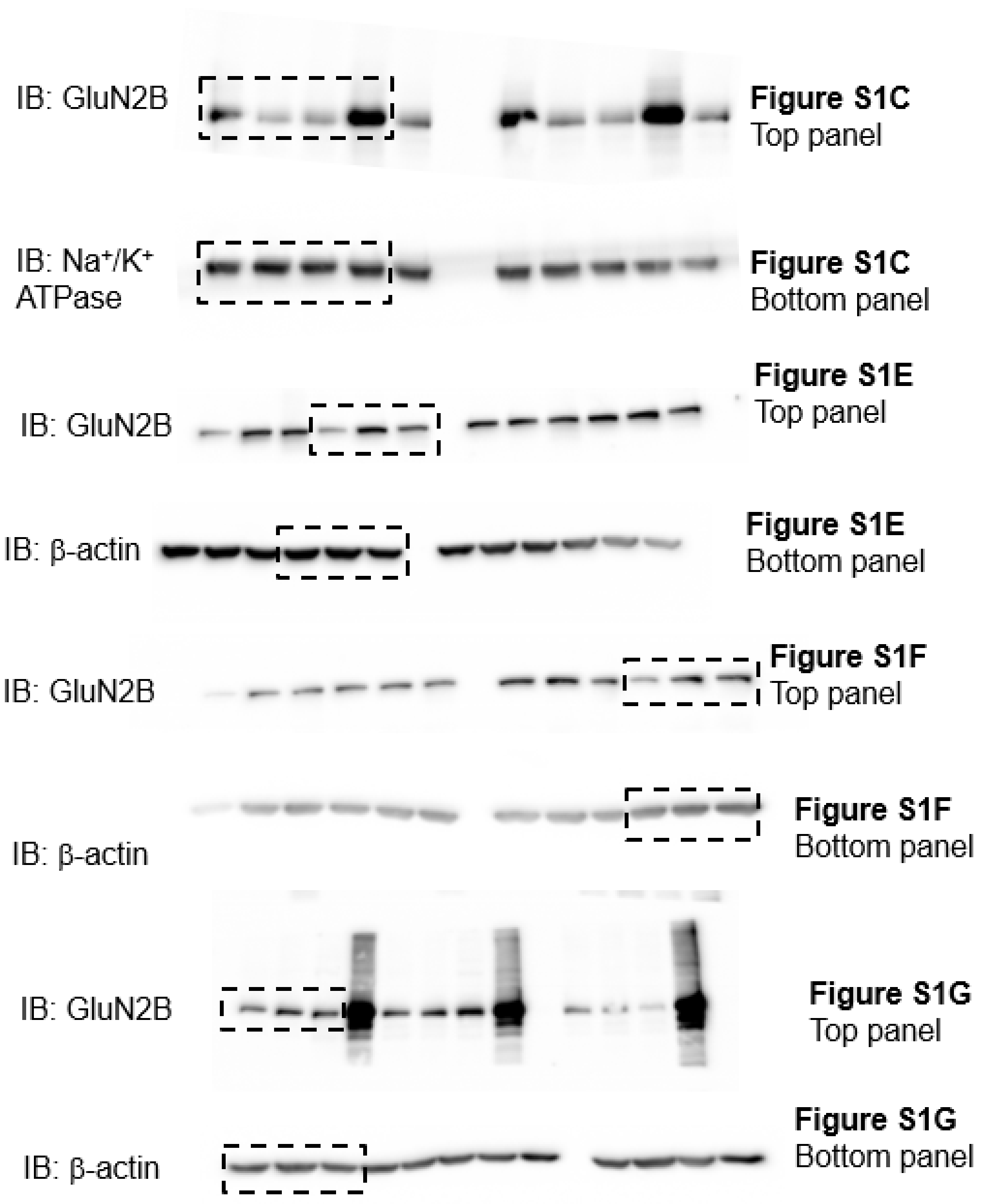

Supplemental Figure 2A/B

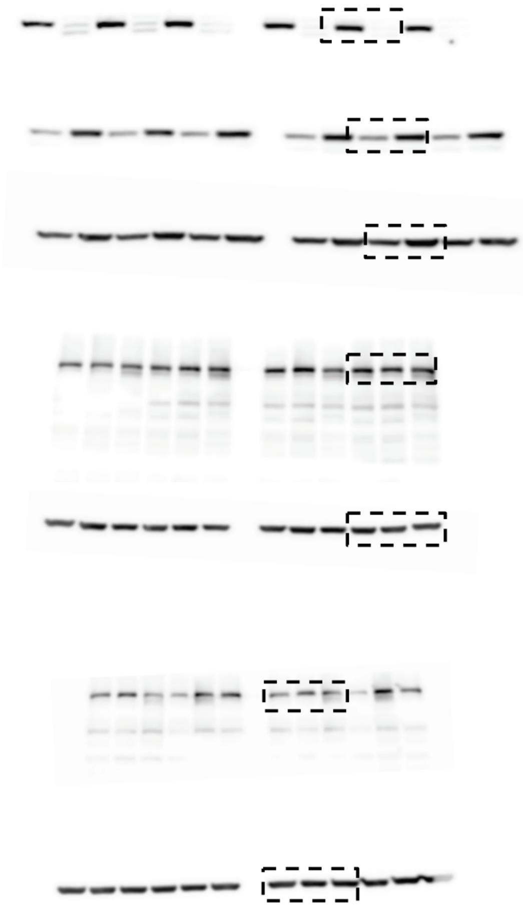

IB: ATG7 — **Figure S2A** Top panel

IB: p62 — **Figure S2A** Middle panel

IB: β-actin — **Figure S2A** Bottom panel

IB: GluN2B — **Figure S2B** Left top panel

IB: β-actin — **Figure S2B** Left bottom panel

IB: GluN2B — **Figure S2B** Right top panel

IB: β-actin — **Figure S2B** Right bottom panel

Supplemental Figure S3A

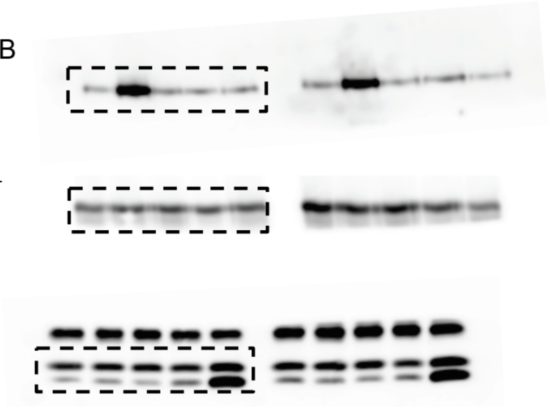

IB: GluN2B — **Figure S3A** Top panel

IB: Na^+/K^+ ATPase — **Figure S3A** Middle panel

IB: LC3b — **Figure S3A** Bottom panel

Supplemental Figure 4C - Input

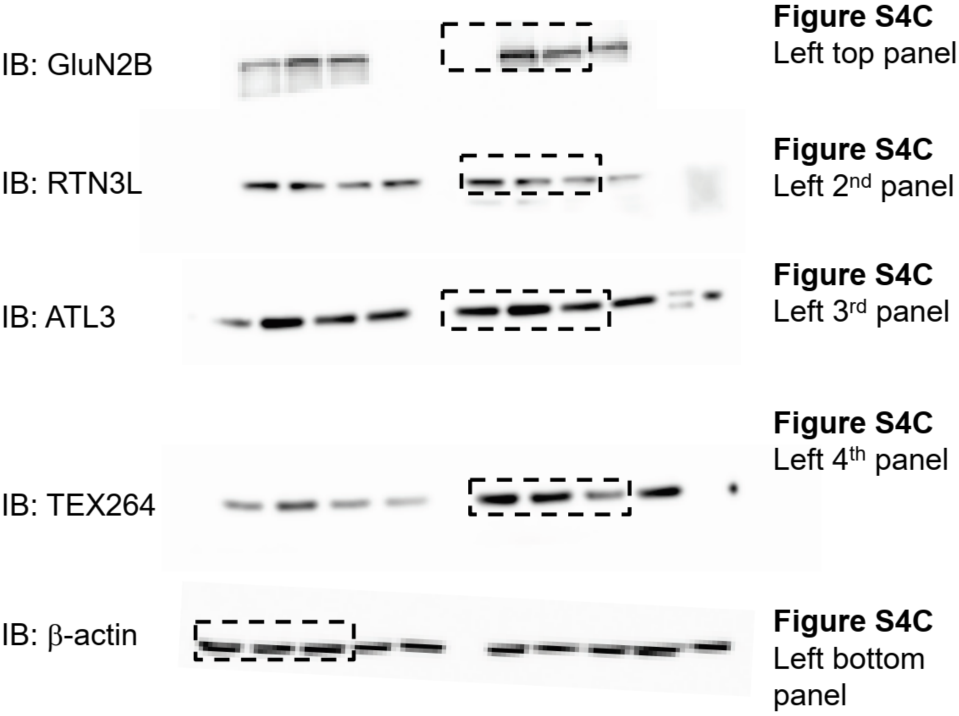

IB: GluN2B — **Figure S4C** Left top panel

IB: RTN3L — **Figure S4C** Left 2nd panel

IB: ATL3 — **Figure S4C** Left 3rd panel

IB: TEX264 — **Figure S4C** Left 4th panel

IB: β-actin — **Figure S4C** Left bottom panel

Supplemental Figure 4C – Pulldown (IP)

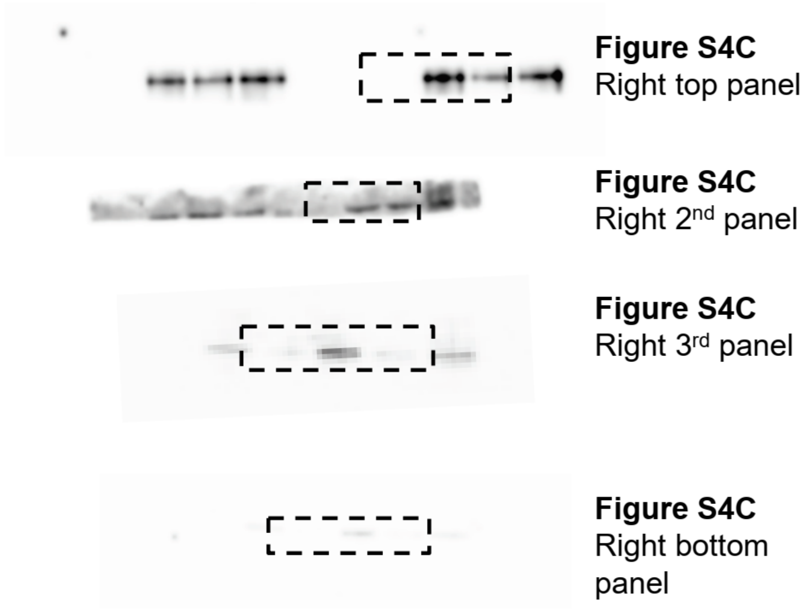

IB: GluN2B — **Figure S4C** Right top panel

IB: RTN3L — **Figure S4C** Right 2nd panel

IB: ATL3 — **Figure S4C** Right 3rd panel

IB: TEX264 — **Figure S4C** Right bottom panel

Supplemental Figure S4D

IB: GluN2B

Figure S4D
Top panel

IB: RTN3L

Figure S4D
Middle panel

IB: β-actin

Figure S4D
Bottom panel

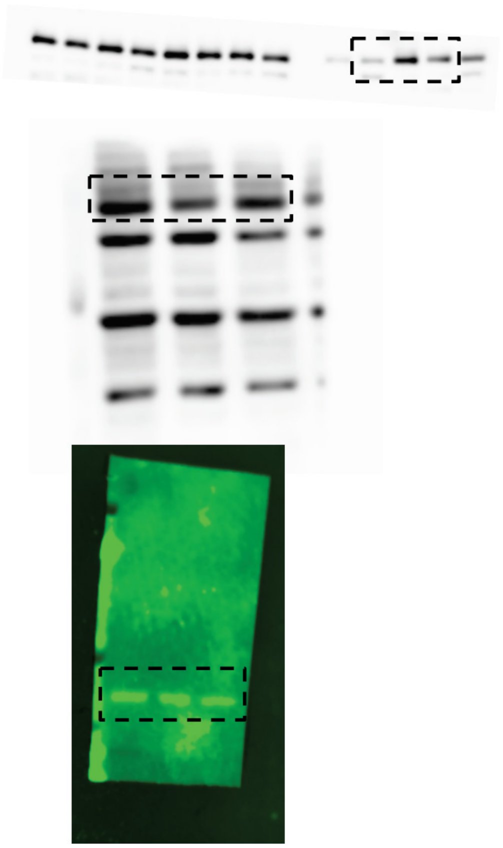

Supplemental Figure S5: Original Western blot images
